# Extended Antimicrobial Profile of Chromone–Butenafine Hybrids

**DOI:** 10.3390/molecules30142973

**Published:** 2025-07-15

**Authors:** Francesca Bonvicini, Lisa Menegaldo, Rebecca Orioli, Federica Belluti, Giovanna Angela Gentilomi, Silvia Gobbi, Alessandra Bisi

**Affiliations:** 1 Department of Pharmacy and Biotechnology, Alma Mater Studiorum-University of Bologna, Via Massarenti 9, 40138 Bologna, Italy; giovanna.gentilomi@unibo.it; 2 Department of Chemistry “G. Ciamician”, Alma Mater Studiorum-University of Bologna, Via Gobetti 85, 40129 Bologna, Italy; lisa.menegaldo@studio.unibo.it; 3 Department of Pharmacy and Biotechnology, Alma Mater Studiorum-University of Bologna, Via Belmeloro 6, 40126 Bologna, Italy; rebecca.orioli3@unibo.it (R.O.); federica.belluti@unibo.it (F.B.); silvia.gobbi@unibo.it (S.G.); 4 Microbiology Unit, IRCCS Azienda Ospedaliero-Universitaria di Bologna, Via Massarenti 9, 40138 Bologna, Italy

**Keywords:** chromone, butenafine, antimicrobial properties, hemolytic activity, cytotoxicity

## Abstract

Fungal infections are recognized as a global health issue, in particular considering the spread of different forms of resistance to the commonly used antifungal drugs and their involvement in the occurrence of co-infections in hospitalized and immunocompromised patients. In this paper, a small series of hybrid compounds were designed and synthesized by linking the privileged chromone and xanthone scaffolds, endowed with recognized antimicrobial potential, to the tert-butylbenzylamino portion of the antifungal drug butenafine, through selected linkers. The results showed for the xanthone-based compound **3** a promising activity towards *C. auris*, *C. tropicalis*, and *C. neoformans*, for which a high degree of resistance is commonly observed, together with a significant antibacterial potency towards Gram-positive bacteria, such as *S. aureus*. Considering that compound **3** displayed favorable selectivity and therapeutic indexes (9.1 and >16, respectively), it appeared as a valuable prototype, deserving further hit-to-lead optimization.

## 1. Introduction

**Figure 1:**
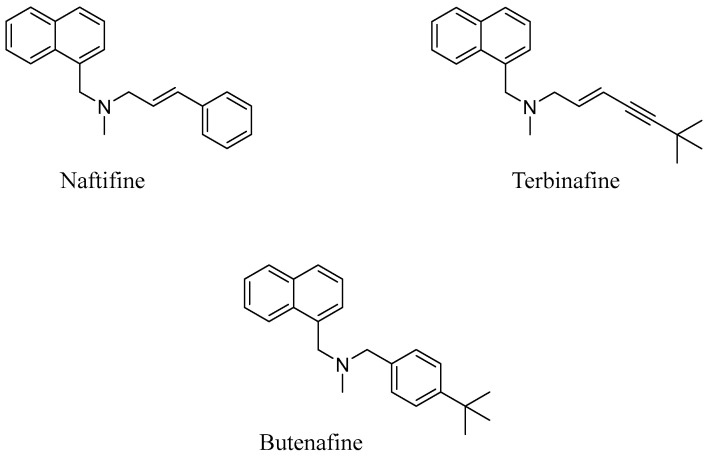
Allylamine- and benzylamine-based antifungal drugs.

Fungal infections (FIs) represent a global health problem, often leading to high morbidity and mortality rates, particularly for patients with impaired immune defenses [1]. FIs can range in severity, from dermatophytosis to superficial infections affecting skin or nails, to invasive fungal infections (IFIs), due to fungi spreading in deep tissues. IFIs often lead to prolonged illness and could become life-threatening, depending on both the disease-causing agent and the infection site, and on host-related factors, such as age and health status [2]. Over the last years, an increase in the number of IFIs, mainly caused by yeasts of the genera Candida and Cryptococcus, has been noticed, owing to the emergence of resistance towards structurally unrelated antifungal drugs and to the increase in the number of immunocompromised or hospitalized patients [3]. In this regard, the occurrence of nosocomial infections often involves different microbial species, and fungal and bacterial co-infections are particularly serious. Actually, the eradication of FIs requires different strategies with respect to those applied to treat bacterial infections, as fungi are eukaryotic organisms, and the search for new active and selective drugs to overcome resistance appears more challenging due to the similarity between fungal and mammalian cells. The main differences relate to the composition of the membrane and cell wall, and the mechanisms of action of available antifungal agents are mainly focused on these molecular targets [4,5,6]. In this respect, drugs belonging to allylamines/benzylamines, azoles, and polyenes were designed to affect the synthesis or to directly bind to ergosterol, the main component of the fungal cell membrane, not found in mammalian cells, and showed the ability to effectively suppress fungal cell growth with negligible effects on host organism. In particular, allylamines, such as naftifine, terbinafine, and the related drug butenafine (*N*-4-tert-butylbenzyl-*N*-methyl-1-naphtalenemethylamine hydrochloride), the only benzylamine-based compound, displayed a broad-spectrum topical antifungal activity and excellent therapeutic efficacy in treating human dermatomycoses (Figure 1) [7].

The mechanism of action of butenafine relies on its ability to inhibit sterol biosynthesis, leading to a depletion of ergosterol, the essential lipid constituent of the fungal membrane, responsible for the regulation of its fluidity, biogenesis, and functions. Its molecular target has been identified as the enzyme squalene epoxidase [8]. Thus, by preventing squalene epoxidation, an accumulation of squalene is observed, and an alteration in membrane function, culminating in fungal death, finally occurs. Notably, butenafine provides a long-lasting antifungal activity and, unlike imidazole and triazole antifungals, does not interact with cytochrome P450-dependent enzymes, and is unable to induce drug-drug interactions-mediated toxicity [9]. Point mutations in the gene of squalene epoxidase have been reported as responsible for increasing resistance to butenafine and related compounds, underlining the need for new antifungal drugs able to face fungal infections engaging multiple targets.

The hybridization of biologically active molecules represents a promising concept in rational drug design, and is based on the identification of pharmacophoric sub-units, independently acting on distinct pharmacological targets, which are blended in a single molecular entity [10,11]. The hybrid drug should then maintain the activities of the selected pharmacophores, featuring a multiple mode of action, reducing the likelihood of drug-drug interactions and the propensity to induce resistance compared to the parent drugs, showing particular efficacy in tackling multifactorial diseases. In this context, natural products and naturally derived compounds, often endowed with multiple pharmacological actions, represent validated privileged scaffolds [12], which could be purposely functionalized in order to address their therapeutic potential in a specific biological field.

Chromone and xanthone are oxygen-containing heterocycles bearing a benzo--pyrone ring, widely distributed in naturally occurring products and endowed with various bioactivities, such as antimalarial, antimicrobial, antioxidant, antiproliferative, antitumor, antiallergic, and anti-inflammatory [13,14]. Notably, the synthetically affordable chromone ring system, a key fragment of xanthone itself and several flavonoids, has emerged as particularly suitable for developing new therapeutic agents upon appropriate structural modification. The hybridization of these oxygenated privileged scaffolds with a peculiar fragment of butenafine could then lead to new molecules endowed with an improved antimicrobial profile.

## 2. Design

**Figure 2:**
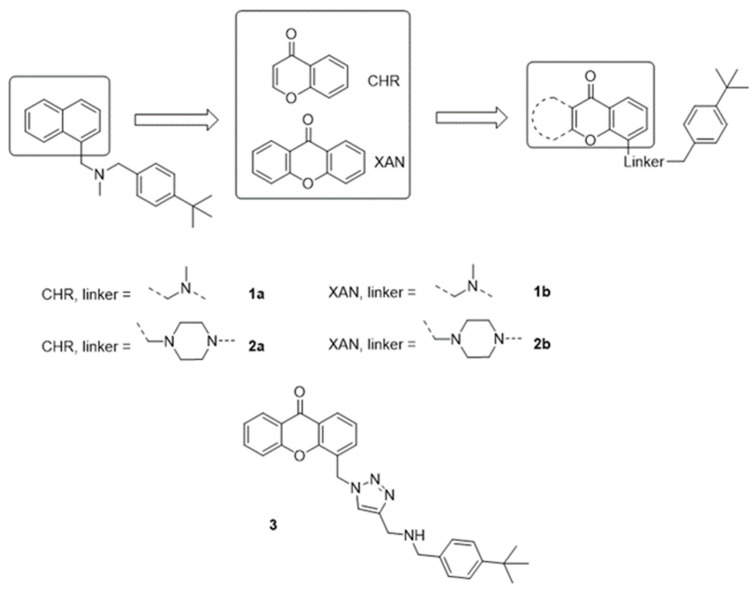
Design of chromone- and xanthone-based prototypes **1a,b, 2a,b**, and **3**.

Some years ago, in a previous paper, our research group designed a small series of xanthone-based derivatives bearing the side chains found in butenafine and naftifine, obtaining compounds endowed with fair antifungal activity [15]. In this work, further prototypes were designed and synthesized, combining the chromone and xanthone scaffolds with the peculiar 4-*tert*-butylbenzylamine portion of butenafine, for a deeper evaluation of the possible role of the γ-pyrone ring in antifungal and antimicrobial activities. In particular, the naphthalene of butenafine was replaced by a chromone core, and the *tert*-butylbenzylamino pharmacophore was introduced at the 8-position of the core structure (compound **1a**, Figure 2), and the previously reported xanthone-based butenafine analogue **1b** was resynthesized to be evaluated in the new assay conditions. Compared to the lead butenafine, the above analogues retained both the planar configuration of the polycyclic scaffold and the insertion position of the substituent. The inclusion of two oxygen atoms in the core structure results in a slight decrease in the overall lipophilicity of the molecules, but introduces the possibility of establishing hydrogen bonds with the potential target. The side chains of **1a** and **1b** were then modified with the introduction of a piperazine in place of the *N*-methyl group while retaining the 4-*tert*-butylbenzyl fragment, thus introducing an additional basic center (**2a-b**, Figure 2). Indeed, piperazine is considered a valuable privileged core in medicinal chemistry, and numerous antibacterial agents bearing this cyclic diamine have been developed [16]. Moreover, piperazine has recently been demonstrated to be responsible for a strong antifungal activity against several species of Candida, maybe due to its capability of interacting with some key proteins involved in cell wall production [17], setting the stage for a possible synergistic effect of the newly designed compounds **2a-b**. Finally, by exploiting the azide-alkyne Huisgen cycloaddition (click chemistry) approach, a xanthone-based derivative was designed, as an analogue of **1b**, in which the side chain was further modified and extended with the introduction of a 1,2,3-triazole ring between the xanthone and the *tert*-butylbenzylamino group (**3**, Figure 2). Indeed, some 1,2,3-triazole hybrids with broad antifungal and antibacterial activities have been reported in the literature [18,19,20] along with the well-known azole antifungal compounds, which are well-known drugs used to combat IFIs. The new prototypes were evaluated against a panel of laboratory and clinical isolates of yeasts and bacteria.

## 3. Results and Discussion

### 3.1. Chemistry

**Scheme 1:**
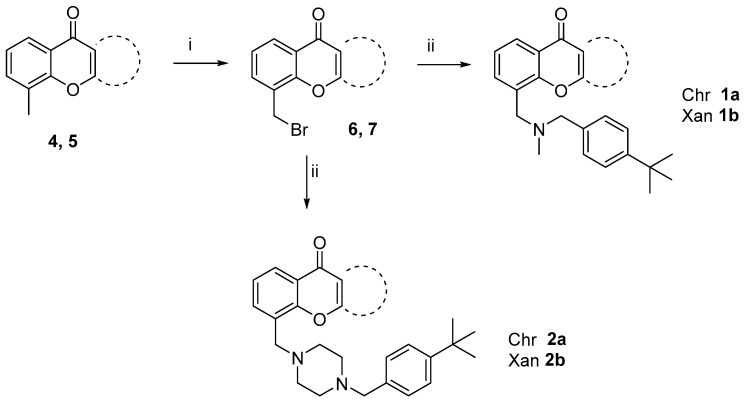
Preparation of compounds **1-2a,b** starting from **4** [21] and **5** [22]. Reagents and conditions: (i) NBS, BPO, CCl_4_, hν, reflux 4 h, 65%; (ii) 1-(4-(*tert*-butyl)phenyl)-N-methylmethanamine [24], 27% (**1a**) and 51% (**1b**) or 1-(4-(tert-butyl)benzyl)piperazine [25], 36% (**2a**) and 17% (**2b**), TEA, toluene, reflux, 8 h.

**Scheme 2:**
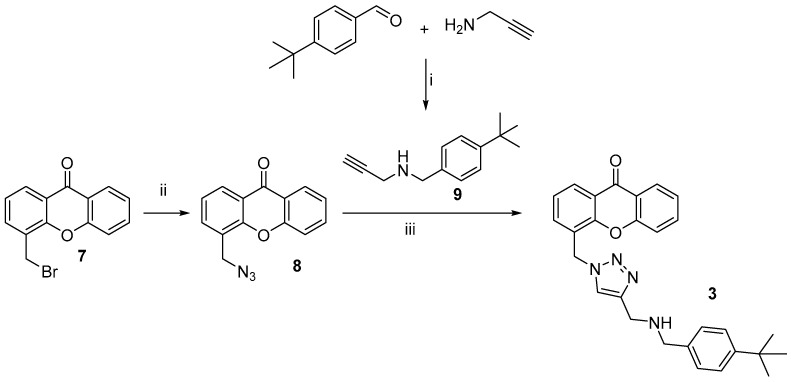
Preparation of compound **3**. Reagents and conditions: (i) EtOH, r.t. 4 h, NaBH_4_, 0 °C-r.t. 18 h, 84%; (ii) NaN_3_, DMSO, 2 r.t. 4 h, 81%; (iii) TEA, CuSO_4_ decahydrate, Na-ascorbate, r.t. 24 h, 14%.

The synthetic strategy to obtain compounds **1-2 a,b** and **3** is depicted in Scheme 1 and Scheme 2.

8-Methyl-4*H*-chromen-4-one **4** and 4-methyl-9*H*-xanthen-9-one **5**, prepared as previously reported [21,22] were subjected to radical bromination by using *N*-bromosuccinimide and benzoyl-peroxide. The bromomethyl intermediates **6** and **7** [23] were then reacted with 1-(4-(*tert*-butyl)phenyl)-*N*-methylmethanamine, in turn obtained by a classical reductive amination between methylamine and 4-(*tert*-butyl)benzaldehyde [24], to obtain the final compounds **1a,b**, or with 1-(4-(*tert*-butyl)benzyl)piperazine, prepared by alkylation of piperazine with 1-(4-(tert-butyl)benzyl)bromide [25], to give **2a,b** (Scheme 1). Compound **3** was obtained starting from **7**, upon treatment with sodium azide to give **8**, followed by azide-alkyne cycloaddition with *N*-(4-(tert-*butyl*)benzyl)prop-2-yn-1-amine **9**, with CuSO_4_ and sodium-ascorbate, to generate, in situ, the copper (I) salt required as catalyst, as outlined in Scheme 2.

### 3.2. Biological Evaluations

The biological properties of the chromone- and xanthone-based prototypes were evaluated in vitro against a panel of laboratory and clinical strains of yeasts and bacteria. The study also included an assessment of the cytotoxicity towards human fibroblasts (HEL 299 CCL-137) and the measurement of the hemolytic activity on human red blood cells (hRBCs), thus obtaining a complete overview on the in vitro antimicrobial effectiveness and safety of the compounds. Finally, assays were performed to investigate their mechanism of action.

#### 3.2.1. In Vitro Antimicrobial Activity

**Figure 3:**
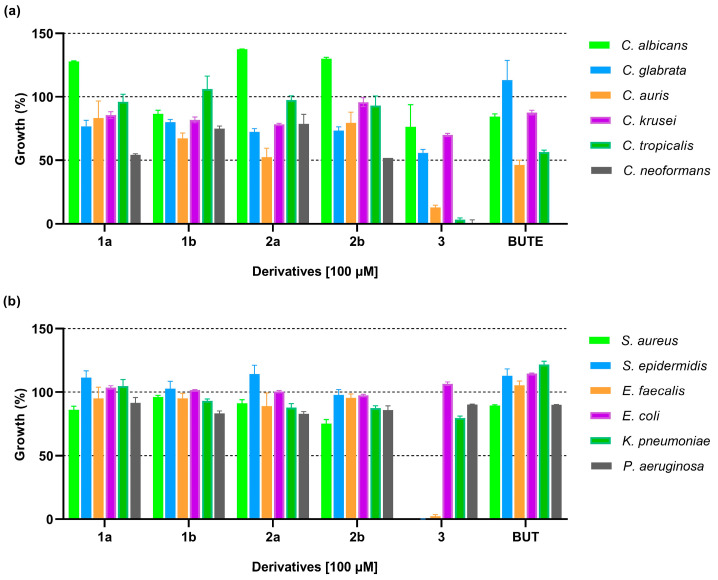
Inhibitory activity of the chromone- and xanthone-based prototypes against fungal growth (**a**) and bacterial growth (**b**). Percentage values are relative to the positive growth control. Data are expressed as mean value and standard deviation.

The antimicrobial activity of the chromone- and xanthone-based prototypes was measured using a standardized microdilution method [26,27] in compliance with the Clinical and Laboratory Standard Institute (CLSI) guidelines [28]. The reference drug controls, fluconazole and gentamicin, were included in the assays, and the minimum inhibitory concentration (MIC) values are detailed in Table S1. Figure 3 displays the inhibitory effects of the compounds tested at a concentration of 100 μM.

Some general remarks can be drawn from data reported in Figure 3a: a moderate inhibitory activity (<50% of growth reduction compared to the control) was observed for derivatives **1a** and **2b** on *C. neoformans*, and for derivative **2a** on *C. auris*. Remarkably, a high inhibitory activity was measured for derivative **3**, which proved to be effective to different extents against several microorganisms. Indeed, while this compound displayed moderate activity against *C. albicans*, *C. glabrata*, and *C. kruzei*, it exhibited a potent inhibitory effect against clinically relevant fungi such as *C. auris*, *C. tropicalis*, and *C. neoformans.* This finding is of particular interest because the non-*albicans Candida* species exhibit varying levels of resistance to commonly used antifungals, thus evading present therapeutic options; for instance, the resistance profile of *C. auris* includes strains that display resistance to polyenes, azoles, and echinocandins, with about 90% resistant to fluconazole [29]. The widespread use of this antifungal agent has also led to the frequent identification of fluconazole-resistant strains, which are responsible for treatment failures in Cryptococcal meningitis [30].

Concerning the antibacterial properties of the compounds (Figure 3b), the xanthone-based derivative **3** proved to be active against the three Gram-positive species, while Gram-negative resulted as completely resistant, possibly indicating a different interaction and uptake of the compound between the multilayered structures of the cell wall of Gram-positive and Gram-negative bacteria.

In the present research, the inhibitory potential of butenafine hydrochloride, the first and only agent in the benzylamine class of antifungals, was also investigated. This compound is a recognized squalene epoxidase inhibitor, effective in suppressing the biosynthesis of ergosterol [31] completely interfering with the growth of *C. neoformans* (<1%) and inactive or moderately active against the other yeasts and bacteria. These results are confirmed by data from the literature, as the activity of butenafine hydrochloride is reported for filamentous fungi rather than yeasts [7], and there is no evidence for its activity against bacteria. Indeed, the butenafine hydrochloride cream (1%), sold under the brand names Lotrimin Ultra and Mentax, is indicated for the topical treatment of tinea infections.

Considering that the ability of butenafine derivatives to act as antibacterial agents has not yet been described, experiments were designed to define the selectivity of the compounds as well as their mechanism of action on bacterial cells; information on their mechanism of action could be helpful to ensure the efficacy and safety of this new class of antimicrobial agents.

#### 3.2.2. Selectivity of Action

**Figure 4:**
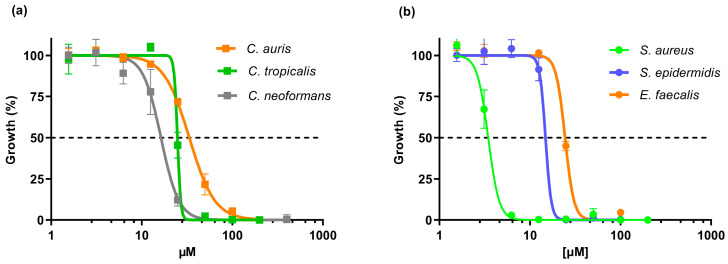
Dose–response curves of derivative **3** against fungal (**a**) and bacterial (**b**) species. Curves were obtained by using a nonlinear regression equation and a variable slope model (log(inhibitor) vs. normalized response–variable slope. The IC_50_ values are represented by the x-coordinates corresponding to the half-maximum growth (dotted lines set at y = 50%) (GraphPad Prism version 9.4.1 for Windows).

To delve into the antimicrobial activity of derivative **3**, IC_50_ values were measured on the susceptible fungal and bacterial species, and these data were discussed in the light of the CC_50_ values and hemolytic activity determined in human cells. Thus, experiments were carried out by testing different concentrations of the compound on *C. auris*, *C. tropicalis*, *C. neoformans*, *S. aureus*, *S. epidermidis,* and *E. faecalis.* As shown in Figure 4, the obtained dose–response curves follow a symmetrical sigmoidal shape, indicating a well-defined quantitative relationship between the tested compound and the cellular effects, thus suggesting a target-dependent action. From the analysis, it emerged that compound **3** was highly effective against *C. neoformans*, among the fungal species, with an IC_50_ value of 16.3 µM, and against *S. aureus*, among the bacterial strains, with an IC_50_ value of 3.5 µM.

Likewise, the CC_50_ value of derivative **3** was defined on HEL 299 cells, measuring 32.3 µM; the selectivity indexes (SIs) for the susceptible strains are reported in Table 1. Remarkably, favorable SI values (>1) were measured for all species, with the only exception of *C. auris*. The minimum hemolytic concentration (MHC) was also calculated on hRBCs, and considering that derivative **3** did not affect cellular membranes even at 100 µM, the therapeutic indexes (TIs) were rated > 1, ensuring the systemic safety of this prototype. Table S2 reports the data of the cytotoxicity and hemolytic activities of the chromone- and xanthone-based prototypes **1a,b** and **2a,b**.

#### 3.2.3. Combined Effect with Derivative **3** and Colistin

To shed light on the different potency of derivative **3** against Gram-positive and Gram-negative species, as well as to investigate the mechanism of action of the compound in bacterial cells, a colistin association assay was performed in *E. coli* by combining the outer membrane (OM) damaging antibiotic colistin, at a sub-inhibitory concentration, with derivative **3**. Remarkably, the activity of the compound was completely restored with an MIC value of 6.25 µM, as measured for *S. aureus*, proving the ability of this active prototype to also affect Gram-negative bacteria, once passing through the OM.

#### 3.2.4. Leakage of Intracellular Contents

**Figure 5:**
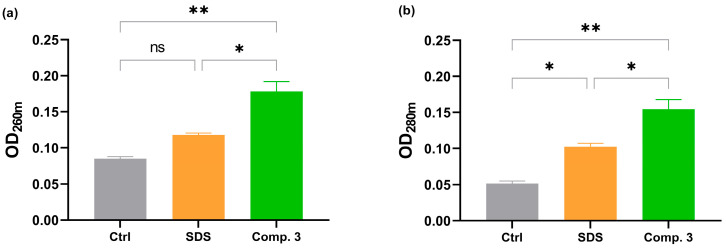
Effects of derivative **3** (12.5 µM) on intracellular nucleic acid ((**a**) OD 260 nm) and protein ((**b**) OD 280 nm) leakage in *S. aureus* ATCC 25923. * *p* ≤ 0.05, ** *p* ≤ 0.01 (as determined by Ordinary One-Way ANOVA for multiple comparisons, GraphPad Prism version 9.4.1 for Windows).

The activity of the xanthone-based prototype **3** on the membrane of *S. aureus* was investigated by incubating the bacterial suspension at 37 °C for 4 h in PBS containing **3** at MIC × 2. Subsequently, the UV-absorbing material released from the cells was spectrophotometrically measured as proof of the increased membrane permeability. The presence of nucleic acids and proteins (Figure 5a,b, respectively) in the supernatants of the treated cells indicated that compound **3** affected *S. aureus* membrane integrity. It has been reported that butenafine hydrochloride interacts with both the hydrophilic and hydrophobic domains of membrane phospholipids, and it is readily incorporated into liposomes, increasing their fluidity and permeability [9]. Taking into account this scientific evidence, it is possible to speculate a similar mechanism of action for the bioactive derivative **3**.

### 3.3. Inhibition of Yeast-to-Hyphae Morphological Transition

**Figure 6:**
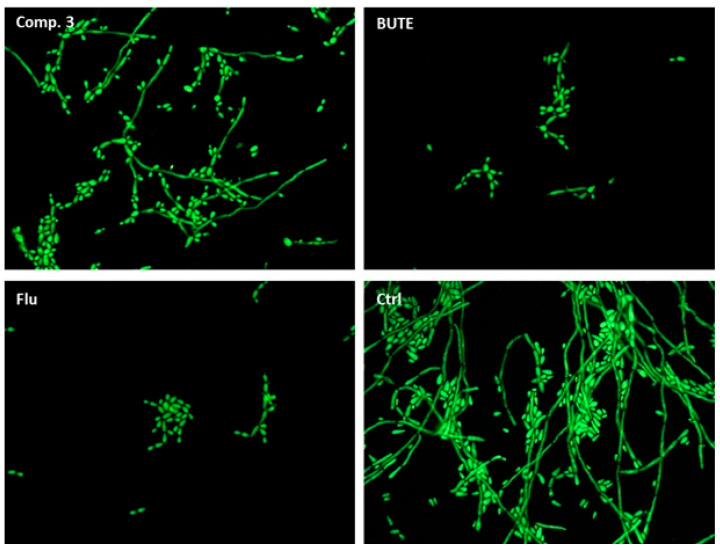
Microscopy images of *C. tropicalis* with SYTO9 collecting fluorescence at 525 nm (green) and with original 40× magnification. Fungal cells were incubated with derivative **3**, butenafine hydrochloride, and fluconazole at IC_50_ values, and in regular medium as positive control.

A key virulence factor expressed by some Candida species that significantly contributes to their pathogenicity is the transition of yeast cells to filamentous cells. The formation of a germ tube and mycelium is an essential process for host tissue invasion, damage to mucosal epithelia, escape from host immune cells, and blood dissemination. The ability of the xanthone-based **3** to weaken fungal virulence was investigated in *C. tropicalis*. For this purpose, *C. tropicalis* was cultured for 2 h in an RPMI-1640 medium supplemented with serum at 10% and containing derivative **3**, butenafine hydrochloride, and fluconazole at IC_50_ values, and then spotted on glass slides and imaged after staining with the cell-permeant, fluorogenic nucleic acid stain SYTO9. Fluorescence images in Figure 6 clearly reveal that butenafine hydrochloride and fluconazole led to a considerable reduction in fungal cells, together with an almost complete absence of hyphae forms, while the prototype **3** only interfered with the yeast–hyphal transition, resulting in a visible reduction in the number of hyphae compared to untreated *C. tropicalis* cells.

### 3.4. In Vitro DNA Binding Interactions

**Figure 7:**
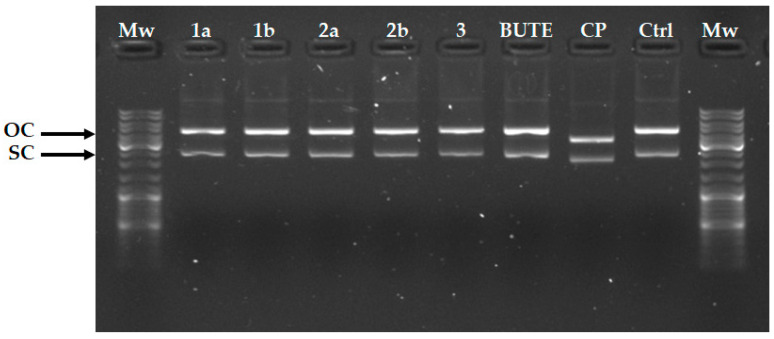
Gel electrophoresis analysis of the plasmid DNA (pmaxGFP of 3527 bp) incubated with 100 µM of the compounds, butenafine hydrochloride (BUTE), and cisplatin (CP). MW (Gene Ruler DNA ladder mix); Ctrl (pmaxGFP) appears in two forms, the open circular form (OC), and the supercoiled form (SC). The incubation with the derivatives did not affect the band pattern after the electrophoresis run.

All the chromone- and xanthone-based prototypes were investigated to ascertain their ability to interact with DNA. For this purpose, a gel electrophoresis analysis was performed after incubating 100 µM of the compounds and 200 ng of plasmid DNA at 37 °C for 2 h. Figure 7 shows that, unlike the reference cisplatin, the compounds did not alter the electrophoretic mobility of the plasmid DNA and exhibited no DNA-cleaving activity. Indeed, no differences were observed in the pattern of bands between the DNA control and the treated samples, both in the open circular and the supercoiled DNA forms. Focusing on derivative **3**, the newly identified antimicrobial agent, it is possible to speculate that its activity on Gram-positive species is not related to bacterial DNA damage but to the inhibition of bacterial replication.

## 4. Materials and Methods

### 4.1. Chemistry

General Methods. All chemicals were purchased from Aldrich Chemistry, Milan (Italy), or Alfa Aesar, Milan (Italy), and were of the highest purity. The selected solvents were of analytical grade. Thin layer chromatography (TLC) on precoated silica gel plates (Merck Silica Gel 60 F254, Darmstadt, Germany) was applied to monitor reaction progress, and then visualized with a UV254 lamplight (Spectroline, New York, NY, USA). Compounds purifications were performed by flash chromatography on silica gel columns (Kieselgel 40, 0.040–0.063 mm, Merck, Darmstadt, Germany). ^1^H NMR spectra for the intermediate compounds were recorded on a Varian Gemini spectrometer working at 400 MHz, while for the final compounds ^1^H NMR and ^13^C NMR spectra were recorded on a Bruker spectrometer working at 600 MHz and at 150 MHz, respectively, in CDCl_3_ and (CD_3_)_2_CO solutions unless otherwise indicated. Chemical shifts (δ) were reported as parts per million (ppm) values relative to tetramethylsilane (TMS) as internal standard; coupling constants (*J*) are reported in Hertz (Hz). Standard abbreviations were used for indicating spin multiplicities: s (singlet), d (doublet), dd (double doublet), ddd (doublet doublet doublets), t (triplet), or m (multiplet). HRMS spectra were recorded on a Waters XevoG2-XS quadrupole time-of-flight apparatus operating in electrospray mode. UHPLC-MS analyses were run on a Waters ACQUITY ARC UHPLC/MS system, consisting of a QDa mass spectrometer equipped with an electrospray ionization interface and a 2489 UV/Vis detector at wavelengths (λ) 254 nm and 365 nm. The analyses were performed on an XBridge BEH C18 column (10 × 2.1 mm i.d., particle size 2.5 μm) with an XBridge BEH C18 VanGuard Cartridge precolumn (5 mm × 2.1 mm i.d., particle size 1.8 μm), with mobile phases consisting of H_2_O (0.1% formic acid) (A) and MeCN (0.1% formic acid) (B). Electrospray (ES) ionization in positive and negative modes was applied in the mass scan range of 50–1200 Da. Method and gradients used were the following: Generic method. Linear gradient: 0–0.78 min, 20% B; 0.78–2.87 min, 20–95% B; 2.87–3.54 min, 95% B; 3.54–3.65 min, 95–20% B; 3.65–5.73 min, 20% B. Flow rate: 0.8 mL/min. All tested compounds were found to have >95% purity. Compounds were named relying on the naming algorithm developed by CambridgeSoft Corporation (Cambridge, MA, USA) and used in ChemBioDraw Ultra (version 23.0).

**8-(bromomethyl)-4H-chromen-4-one (6).**

8-methyl-4H-chromen-4-one **4**, prepared as previously reported [21] (1.13 g, 7.02 mmol, 1 eq) in CCl_4_, was treated with NBS (1.24 g, 7.02 mmol, 1 eq) and a catalytic amount of benzoyl peroxide (BPO). The mixture was gently heated under light irradiation and followed by TLC. After 4 h, the suspension was hot filtered, cooled, filtered again, and the solvent was removed under reduced pressure. The raw material was then purified by flash chromatography (PE/EtOAC 4.5:0.5), to yield a pale-yellow solid (1.10 g, 65%, mp 187–189 °C). ^1^H NMR: δ 4.72 (s, 2H, CH_2_), 6.39 (d, *J* = 6.0 Hz, 1H, =CH), 7.39 (m, 1H, arom), 7.73 (d, *J* = 5.6 Hz, 1H, =CH), 7.95 (d, *J* = 6.0 Hz, 1H, arom), 8.19 (d, *J* = 9.6 Hz, 1H, arom).

**General procedure for the preparation of the final compounds 1-2a,b.**

A solution of 8-(bromomethyl)-4H-chromen-4-one **6** or 4-(bromomethyl)-9H-xanthen-9-one **7** (prepared as previously reported by us [23]) in toluene (30 mL) was treated with Et_3_N (1 eq), and the selected amine, prepared as reported in the literature. The mixture was heated under reflux for about 8 h, followed by TLC. After cooling at r.t., the suspension was washed with water, dried over Na_2_SO_4_ anhydrous, and evaporated to dryness. The crude was purified by flash chromatography to obtain the final compound.

**8-(((4-(*tert*-butyl)benzyl)(methyl)amino)methyl)-4H-chromen-4-one (1a).**

Using the previous procedure and starting from **6** (0.5 g, 2.08 mmol, 1 eq), (Et)_3_N (0.3 mL, 2.08 mmol, 1 eq), and 1-(4-(*tert*-butyl)phenyl)-*N*-methylmethanamine, prepared as reported in the literature [24] (0.37 g, 2.08 mmol, 1 eq), **1a** was obtained as a yellow solid. The crude was purified by flash chromatography (toluene/acetone 4.75:0.25) to yield 0.20 g (27%, mp 93–95 °C). ^1^H NMR: δ 1.30 (s, 9H, (CH_3_)_3_), 2.24 (s, 3H, CH_3_), 3.58 (s, 2H, CH_2_), 3.73 (s, 2H, CH_2_), 6.30 (d, *J* = 6.0 Hz, 1H, Ar), 7.29 (d, *J* = 8.3 Hz, 2H, Ar), 7.32–7.37 (m, 3H, Ar), 7.79 (d, *J* = 6.1 Hz, 2H, Ar), 8.09 (dd, *J* = 8.0, 1.7 Hz, 1H, Ar). ^13^C NMR: δ 31.34 (3C, C(CH_3_)_3_), 34.37 (Al), 42.45 (Al), 54.39 (Al), 61.95 (Al), 112.63 (Ar), 124.36 (Ar) 124.69 (Ar), 124.76 (Ar), 125.04 (2C, Ar), 128.55 (2C, Ar), 128.67 (Ar), 134.41 (Ar), 135.71 (Ar), 149.89 (Ar), 154.73 (Ar), 154.97 (Ar), 177.66 (1C, C=O). HRMS (*m*/*z*): [M + H]^+^ calc. for C_22_H_25_NO_2_ 335.18853; found 336.1957.

**4-(((4-(*tert*-butyl)benzyl)(methyl)amino)methyl)-9H-xanthen-9-one (1b).**

Using the previous procedure and starting from **7** (0.4 g, 1.38 mmol, 1 eq), (Et)_3_N (0.2 mL, 1.38 mmol, 1 eq), and 1-(4-(*tert*-butyl)phenyl)-*N*-methylmethanamine (0.25 g, 1.38 mmol, 1 eq), **1b** was obtained as a yellow solid. The compound was purified by flash chromatography (PE/DCM 4:1) to yield 0.27 g (51%, oil). ^1^H NMR: δ 1.33 (s, 9H, (CH_3_)_3_), 2.32 (s, 3H, CH_3_), 3.67 (s, 2H, CH_2_), 3.91 (s, 2H, CH_2_), 7.34–7.42 (m, 7H, Ar), 7.71 (ddd, *J* = 8.6, 7.1, 1.7 Hz, 1H, Ar), 7.89 (d, *J* = 7.3 Hz, 1H, Ar), 8.26 (dd, *J* = 7.9, 1.8 Hz, 1H, Ar), 8.33 (dd, *J* = 7.9, 1.8 Hz, 1H, Ar). ^13^C NMR: δ 31.55 (3C, C(CH_3_)_3_), 34.65 (Al), 42.70 (Al), 54.64 (Al), 62.32 (Al), 118.22 (Ar), 121.76 (Ar), 121.99 (Ar), 123.59 (Ar), 124.05 (Ar), 125.40 (2C, Ar), 125.65 (Ar), 125.77 (Ar), 126.80 (Ar), 127.05 (Ar), 128.95 (2C, Ar), 134.78 (Ar), 136.01 (Ar), 150.23 (Ar), 154.56 (Ar), 156.08 (Ar), 177.57 (C=O). HRMS (*m*/*z*): [M + H]^+^ calc. for C_26_H_27_NO_2_ 385.2042; found 386.2111.

**8-((4-(4-(tert-butyl)benzyl)piperazin-1-yl)methyl)-4H-chromen-4-one (2a).**

Using the previous procedure and starting from **6** (0.55 g, 2.08 mmol, 1 eq), (Et)_3_N (0.3 mL, 2.08 mmol, 1 eq), and 1-(4-(*tert*-butyl)benzyl)piperazine, prepared as previously reported [25] (0.49 g, 2.08 mmol, 1 eq), **2a** was obtained. The crude was purified by flash chromatography (toluene/acetone 4:1), to obtain 0.29 g of pale-yellow solid (36%, mp 102–104 °C). ^1^H NMR: δ 1.31 (s, 9H, (CH_3_)_3_), 2.30–2.87 (m, 8H, H-piperazine), 3.53 (s, 2H, CH_2_), 3.79 (s, 2H, CH_2_), 6.34 (d, *J* = 6.0 Hz, 1H, Ar), 7.24 (d, *J* = 8.2 Hz, 2H, Ar), 7.31–7.34 (m, 2H), 7.37 (t, *J* = 7.7 Hz, 1H, Ar), 7.73 (dd, *J* = 7.4, 1.7 Hz, 1H, Ar), 7.87 (d, *J* = 6.0 Hz, 1H, Ar). ^13^C NMR: 31.51 (3C, C(CH_3_)_3_), 34.62 (Al), 52.99 (Al), 55.57 (Al), 62.64 (Al), 112.98 (Ar), 124.90 (Ar), 124.92 (Ar), 125.06 (Ar), 125.30 (2C, Ar), 126.14 (Ar), 127.56 (Ar), 129.23 (Ar), 129.84 (Ar), 134.95 (Ar), 150.30 (Ar), 155.02 (Ar), 155.17 (Ar), 177.96 (C=O). HRMS (*m*/*z*): [M + H]^+^ calc. for C_25_H_30_N_2_O_2_ 390.2307; found 391.2373.

**4-((4-(4-(*tert*-butyl)benzyl)piperazin-1-yl)methyl)-9H-xanthen-9-one (2b).**

Using the previous procedure and starting from **7** (0.38 g, 1.32 mmol, 1 eq), (Et)_3_N (0.19 mL, 1.32 mmol, 1 eq), and 1-(4-(*tert*-butyl)benzyl)piperazine, (0.31 g, 1.32 mmol, 1 eq), **2b** was obtained. The crude was purified by flash chromatography (PE/EtOAc 4:1), to obtain 0.10 g of white solid (17%, mp 98–101 °C). ^1^H NMR: δ 1.30 (s, 9H, (CH_3_)_3_), 2.34–2.77 (m, 8H, H-piperazine), 3.50 (s, 2H, CH_2_), 3.91 (s, 2H, CH_2_), 7.23 (d, *J* = 8.1 Hz, 2H, Ar), 7.29–7.40 (m, 4H, Ar), 7.45–7.54 (m, 1H, Ar), 7.71 (ddd, *J* = 8.6, 7.1, 1.7 Hz, 1H, Ar), 7.80 (dd, *J* = 7.3, 1.7 Hz, 1H, Ar), 8.25 (dd, *J* = 7.9, 1.7 Hz, 1H, Ar), 8.32 (dd, *J* = 7.9, 1.7 Hz, 1H, Ar). ^13^C NMR: δ 31.46 (3C, C(CH_3_)_3_), 34.52 (Al), 53.12 (2C, piperazine), 53.14 (2C, piperazine), 55.50 (Al), 62.75 (Al), 118.07 (Ar), 121.66 (Ar), 121.84 (Ar), 123.53 (Ar), 124.02 (Ar), 125.16 (Ar), 125.66 (3C, Ar), 126.74 (Ar), 127.10 (Ar), 129.07 (2C, Ar), 134.81 (Ar), 135.88 (Ar), 150.02 (Ar), 154.44 (Ar), 155.92 (Ar), 177.41 (C=O). HRMS (*m*/*z*): [M + H]^+^ calc. for C_29_H_32_N_2_O_2_ 440.2463; found 441.2520.

**4-(azidomethyl)-9H-xanthen-9-one (8).**

A solution of **7** (0.5 g, 1.73 mmol, 1 eq) and NaN_3_ (0.14 g, 2.07 mmol, 1.2 eq) in DMSO (20 mL) was stirred at r.t. for 24 h. The slurry was quenched with H_2_O and the solid obtained was collected by filtration to give **8** (0.37 g, 84%), used for the subsequent step without further purification. ^1^H NMR: δ 4.75 (s, 2H, CH_2_N), 7.40–7.43 (m, 3H, arom), 7.58 (d, *J* = 8.0 Hz, 1H, arom), 7.73 (d, *J* = 6.0 Hz, 1H, arom), 7.77 (d, *J* = 5.6 Hz, 1H, arom), 8.37 (d, *J* = 7.6 Hz, 1H, arom). ^13^C NMR: δ 49.85 (CH_2_N), 118.26 (Ar), 121.87 (Ar), 122.28 (Ar), 123.90 (Ar), 124.56 (Ar), 125.00 (Ar), 126.96 (Ar), 127.42 (Ar), 135.11 (Ar), 135.25 (Ar), 154.25 (Ar), 155.89 (Ar), 177.07 (C=O).

***N*-(4-(*tert*-butyl)benzyl)prop-2-yn-1-amine (9).**

A solution of prop-2-yn-1-amine (0.17 g, 3.1 mmol, 1 eq) and 4-(*tert*-butyl)benzaldehyde (0.5 g, 3.1 mol, 1 eq) in EtOH (35 mL) was stirred for 4 h at r.t. and then cooled at 0 °C. NaBH_4_ (0.34 g, 9.3 mmol, 3 eq) was slowly added and the mixture was stirred for 18 h at r.t., quenched with H_2_O, and extracted with DCM. The solvent was evaporated to dryness to obtain 0.59 g of **9** (81%) as colorless oil, used for the subsequent step without further purification. ^1^H NMR: δ 1.33 (s, 9H, (CH_3_)_3_), 2.26 (s, 1H, ≡CH), 3.44 (s, 2H, ≡C-CH_2_), 3.82 (s, 2H, N-CH_2_), 4.67 (s, 1H, NH), 7.83 (d, *J* = 8 Hz, 2H, arom), 7.34 (d, *J* = 10.8 Hz, 2H, arom) [32].

**4-((4-(((4-(tert-butyl)benzyl)amino)methyl)-1H-1,2,3-triazol-1-yl)methyl)-9H-xanthen-9-one (3).**

To a solution of the alkyne **9** (0.5 g, 2.5 mmol, 1 eq) in DMSO, the azide **8** (0.82 g, 3.3 mmol, 1.3 eq) and TEA (0.04 mL, 0.25 mmol, 0.1 eq) were added. A solution of CuSO_4_ decahydrate (0.06 g, 0.1 eq) and sodium ascorbate (0.25 g, 0.5 eq) in H_2_O was prepared and promptly added to the reaction mixture, which was stirred for 72 h at r.t. and then poured into ice. The mixture was extracted with DCM (3 × 40 mL), and the organic layer was washed with H_2_O, dried over Na_2_SO_4_, and evaporated to dryness. The obtained crude product was purified by flash chromatography (toluene/acetone 3.5:1.5) and recrystallized from diethylether to give 0.16 g of **3** (14%, oil). ^1^H NMR: δ 1.29 (s, 9H, (CH_3_) _3_), 3.77 (d, *J* = 10.6, 2H, CH_2_), 3.92 (s, 2H, CH_2_), 5.90 (s, 2H, CH_2_), 7.21 (d, *J* = 7.8 Hz, 2H, Ar), 7.31 (d, *J* = 7.5 Hz, 2H, Ar), 7.37–7.45 (m, 2H, Ar), 7.51–7.59 (m, 2H, Ar), 7.61 (d, *J* = 7.3 Hz, 1H), 7.75 (ddd, *J* = 8.6, 7.1, 1.7 Hz, 1H, Ar), 8.36 (ddd, *J* = 12.1, 8.0, 1.6 Hz, 2H, Ar). ^13^C NMR: δ 29.85 (Al), 31.10 (3C, C(CH_3_)_3_), 31.49 (Al), 34.60 (Al), 48.63 (Al), 118.10 (Ar), 121.82 (Ar), 122.39 (Ar), 124.16 (Ar), 124.75 (Ar), 125.30 (Ar), 125.56 (Ar), 127.03 (Ar), 128.03 (Ar), 128.23 (Ar), 128.78 (Ar), 135.30 (Ar), 135.40 (Ar), 153.78 (Ar), 155.75 (Ar), 176.79 (C=O). HRMS (*m*/*z*): [M + H]^+^ calc. for C_28_H_28_N_4_O_2_ 452.2212; found 453.2265.

### 4.2. Compounds and Reference Drugs

The dry powder of the chromone- and xanthone-based prototypes was dissolved in 100% DMSO at 20 mM, and then diluted with the appropriate medium to achieve the required concentrations. Butenafine hydrochloride, fluconazole, gentamicin, and cisplatin were purchased from Sigma-Aldrich (St. Louis, MO, USA) and used as reference compounds in the biological assays.

### 4.3. Biological Evaluation

#### 4.3.1. Microbial Strains and Growth Conditions

A panel of laboratory and clinical strains of yeasts and bacteria was included in the study: *Candida albicans* (ATCC 10231), *Candida glabrata*, *Candida auris*, *Candida krusei*, *Candida tropicalis*, *Cryptococcus neoformans*, *Staphylococcus aureus* (ATCC 25923), *Staphylococcus epidermidis* (ATCC 12228), *Enterococcus faecalis* (ATCC 29212), *Escherichia coli* (ATCC 25922), *Klebsiella pneumoniae* (ATCC 9591), and *Pseudomonas aeruginosa* (ATCC 2783). The laboratory strains were obtained from the American Type Culture Collection (ATCC, Manassas, VA, USA). The fungal cultures were routinely grown at 37 °C on Sabouraud Dextrose agar plates, and bacterial cultures were grown on 5% blood agar plates.

#### 4.3.2. MIC and IC_50_ Determination

The antimicrobial properties of the compounds and butenafine hydrochloride were determined by a previously established microdilution method [26,27]. In short, fresh colonies obtained on the agar plates were used to prepare microbial suspensions (at optical density of 630 nm) and diluted 1:200 in Mueller–Hinton broth (MH CondaLab, Laboratorios Conda S.A., Madrid, Spain) for the antibacterial assays, and 1:20 in RPMI-1640 medium (Microgen Laboratory Research srl) containing glucose 2%, 0.3% levo-glutamine buffered to pH 7.0 with 0.165 M 3-(N-morpholino)propanesulfonic acid (MOPS) for the antifungal assays. All the compounds were twofold serially diluted and tested in the range of 100–0.78 µM. Positive growth controls (microbial suspension in regular medium) and negative controls (only medium), together with background and solvent controls (DMSO dilutions), were included in the tests. Fungal and bacterial suspensions were also assayed with fluconazole and gentamicin, respectively, as internal drug controls. Range concentrations and results are reported in Table S1. The plates were incubated at 37 °C for 24 h, and the OD_630nm_ was measured. MIC values were defined as the concentration of the compounds inhibiting 90% of microbial growth relative to the positive controls, and IC_50_ values were defined as the concentration giving rise to an inhibition of growth of 50%, obtained from nonlinear regression analysis (GraphPad Prism version 9.4.1, San Diego, CA, USA).

#### 4.3.3. Cell Viability and Proliferation Assay

The HEL 299 cell line, obtained from ATCC (CCL-137) was selected as the model system to investigate the effect of the compounds on nonmalignant mammalian fibroblasts. Briefly, cells were cultured in EMEM (Lonza, Walkersville, MD, USA) supplemented with 10% fetal bovine serum (Microgen Laboratory Research srl), 100 µg/mL penicillin, and 100 µg/mL streptomycin at 37 °C with 5% CO_2_. For experiments, cells were seeded into 96-well plates at 10^4^ cells/well, and incubated at 37 °C for 24 h. Following washes with PBS, cell monolayer was incubated with 100 µL of medium containing the twofold serial dilutions of the compounds in the range of 100–0.78 µM. Cell viability was assessed by a WST8-based assay according to the manufacturer’s instructions (Enhanced cell counting kit 8, Elabscience Bionovation Inc., Houston, TX, USA). After 48 h of incubation, culture medium was removed, the monolayer was washed with PBS, and 100 µL of fresh medium containing 10 µL of WST-8 solution were added. Following 2 h of incubation at 37 °C, the OD_450nm_ was read and data were expressed as the percentage of cell viability relative to the untreated controls. The CC_50_ was obtained on the corresponding dose–response curves generated as previously reported for IC_50_ values.

#### 4.3.4. Hemolytic Activity Assay

The hemolytic activity of the compounds was evaluated as the amount of hemoglobin released by the disruption of human red blood cells (hRBCs). For the experiments, fresh hRBCs, obtained from peripheral blood of anonymous blood donors available for research purposes, were collected by centrifugation, washed with PBS, and resuspended to a final concentration of 4% *w*/*v* hRBCs in PBS. Then, 100 µL of hRBCs suspension and an equal volume of the twofold dilutions (range 100–0.78 µM) of the samples were mixed in a 96-well plate and incubated for 1 h at 37 °C. Finally, the supernatants were spectrophotometrically evaluated at OD_405nm_. Untreated hRBCs (in PBS) and hRBCs incubated with 1% Triton X-100 were employed as negative and positive controls, respectively. The hemolysis percentage was calculated as [OD_405nm_ (sample) − OD_405nm_ (negative control)]/[OD_405nm_ (positive control) − OD_405nm_ (negative control)] × 100. Minimal hemolytic concentrations (MHCs) were defined as the compound concentration causing 10% hemolysis.

### 4.4. In Vitro DNA Cleavage/Mobility Shift Assays

Studies of interactions between the chromone- and xanthone-based prototypes and a plasmid DNA were carried out using agarose gel electrophoresis. Thus, 200 ng of pmaxGFP plasmid, 3527 bps (Lonza, Basel, Switzerland), were incubated with the compounds at 100 μM in Tris-HCl buffer (NaCl 50 mM, Tris-HCl 5 mM, pH 7.20). The mixture was incubated at 37 °C for 2 h, then the reactions were quenched by adding 5 μL of the loading buffer solution and analyzed by 1% agarose gel electrophoresis.

### 4.5. Analysis of the Yeast-to-Hyphae Transition

Suspensions of *C. tropicalis* (5 × 10^5^ cells/mL) were prepared in RPMI culture medium supplemented with 10% FBS and incubated for 2 h at 37 °C with compound **3**, butenafine hydrochloride, and fluconazole at IC_50_ values, in a 48-well flat bottomed microplate. The different fungal morphotypes were observed under a fluorescence microscope after staining with SYTO 9 dye (SYTO 9 Green Fluorescent Nucleic Acid Stain, Thermo Fisher Scientific, Waltham, MA, USA).

### 4.6. Leakage of Intracellular Contents

The loss of DNA/RNA and proteins was determined by means of a previously described protocol [33]. Briefly, an overnight microbial culture was centrifuged at 4500× *g* for 5 min at 4 °C, then washed twice in cold PBS. Cell were incubated at 37 °C with 1 mL of PBS (positive control), 5% SDS (sodium dodecyl sulfate) in PBS (treated control), and derivative **3** at MIC × 2 (12.5 μM). After 4 h, the cell suspensions were centrifuged at 13.400× *g* for 15 min and the supernatants were collected and, respectively, read at 260 nm and 280 nm for the measurements of the nucleic acid and protein contents.

## 5. Conclusions

In this paper, to further explore the favorable role of the γ-pyrone fragment in the design of potential antifungal and antimicrobial agents, a small series of prototypes was designed and synthesized by exploiting the privileged structure of chromone and xanthone scaffolds. In particular, these core structures were connected to the *tert*-butylbenzylamino portion of the antifungal drug butenafine, either directly or through piperazino and 1,2,3-triazole linkers, as synthons frequently observed in antimicrobials. The new prototypes were evaluated against a panel of laboratory and clinical isolates of yeasts and bacteria. The best results were shown for the xanthone-based analogue **3**, endowed with a promising antimicrobial profile, together with remarkable cyto-compatibility. Indeed, regarding antifungal activity, this derivative proved to be effective towards different microorganisms, in particular *C. auris*, *C. tropicalis*, and *C. neoformans*, for which a high degree of resistance is commonly observed. Moreover, the ability of **3** to interfere with the yeast–hyphal transition, inducing a visible decrease in the number of hyphae, was observed in *C. tropicalis*. In addition, the antibacterial activity of compound **3** broadens its antimicrobial profile, as it was able to inhibit the growth of Gram-positive as well as Gram-negative species with the same potency when the OM was permeabilized. As for the mechanism of action, the capability of **3** to affect the plasma membrane was suggested by the leakage of the intracellular bacterial content and indirectly corroborated by the observed inability of the compound to damage or to bind to the bacterial DNA. Interestingly, at antimicrobial concentrations, compound **3** displayed no activity towards human fibroblasts and hRBCs, indicating high target selectivity and good TIs.

Taken together, these data outline the xanthone-based compound **3** as a promising prototype, endowed with an expanded polymicrobial activity compared to butenafine and a significant safety profile, worthy of further investigation in a medicinal chemistry campaign.

## Figures and Tables

**Table 1:** Selectivity of action of derivative **3** on the susceptible microbial species.

Species	IC_50_ ^1^	SI ^2^	MIC ^3^	TI ^4^
*C. tropicalis*	24.8	1.3	50	>2
*C. auris*	33.6	1.0	100	>1
*C. neoformans*	16.3	2.0	25	>4
*S. aureus*	3.5	9.2	6.25	>16
*S. epidermidis*	14.8	2.2	25	>4
*E. faecalis*	24.4	1.3	50	>2

^1^ IC_50_ value is the concentration of the compound giving rise to an inhibition of 50% compared to the drug-free control. ^2^ SI: Selectivity index is calculated as ratio of CC_50_ to IC_50_. ^3^ MIC value is the concentration of the compound inhibiting 90% of the microbial growth compared to the drug-free control. ^4^ Therapeutic index is calculated as ratio of MHC value to the MIC.

## Data Availability

Data is contained within the article. The original contributions presented in this study are included in the article. Further inquiries can be directed to the corresponding authors.

## Supplementary Materials

The following supporting information can be downloaded at: https://www.mdpi.com/article/10.3390/molecules30142973/s1, Table S1: MIC values of the reference drug controls (μg/mL); Table S2: Percentage values of cell viability and hemolytic activity at 100 μM; Figures S1–S13: ^1^H and ^13^C NMR spectra of the final compounds.

## Abbreviations

The following abbreviations are used in this manuscript:

IFIs	Invasive fungal infections
hRBCs	Human red blood cells
MIC	Minimum inhibitory concentration
MHC	Minimum hemolytic concentration
TI	Therapeutic index
OM	Outer membrane
