# Supplementary material for: Extended Antimicrobial Profile of Chromone–Butenafine Hybrids
Source: Molecules. 2025 Jul 15;30(14):2973. doi: 10.3390/molecules30142973 (PMC12300761; doi:10.3390/molecules30142973)
Supplement: Supplementary file 1 [file molecules-30-02973-s001.zip › molecules-3719755-supplementary.pdf]

SUPPORTING INFORMATION

EXTENDED ANTIMICROBIAL PROFILE OF CHROMONE-BUTENAFINE HYBRIDS

Francesca Bonvicini [1]*, Lisa Menegaldo [2], Rebecca Orioli [3], Federica Belluti [3], Giovanna Angela Gentilomi [1,4], Silvia Gobbi [3], and Alessandra Bisi [3]*

[1] Department of Pharmacy and Biotechnology, Alma Mater Studiorum-University of Bologna, Via Massarenti 9, 40138 Bologna, Italy;
[2] Department of Chemistry "G. Ciamician", Alma Mater Studiorum-University of Bologna, Via Gobetti 85, 40129 Bologna, Italy;
[3] Department of Pharmacy and Biotechnology, Alma Mater Studiorum-University of Bologna, Via Belmeloro 6, 40126 Bologna, Italy;
[4] Microbiology Unit, IRCCS Azienda Ospedaliero-Universitaria di Bologna, Via Massarenti 9, 40138 Bologna, Italy;

* Correspondence: alessandra.bisi@unibo.it; (A.B.)

* Correspondence: francesca.bonvicini4@unibo.it; (F.Bonvicini)

Table S1. MIC values of the reference drug controls (μg/mL)

Table S2. Percentage values of cell viability and hemolytic activity at 100 μM

Figures S1-S10. ^{1}H and ^{13}C NMR spectra of the final compounds

Figures S11-S13. ^{1}H and ^{13}C NMR spectra of intermediates **8** and **6**

Table S1. MIC values of the reference drug controls (μg/mL)

Species	Fluconazole[1]	Gentamicin[2]
C. albicans	25	n.d.[3]
C. glabrata	25	n.d.
C. auris	>50	n.d.
C. kruzei	50	n.d.
C. tropicalis	1.56	n.d.
C. neoformans		n.d.
S. aureus	n.d.	0.31
S. epidermidis	n.d.	0.31
E. faecalis	n.d.	>2.5
E. coli	n.d.	0.62
K. pneumoniae	n.d.	0.62
P. aeruginosa	n.d.	0.156

[1] tested range concentration: 50 – 0.31 μg/mL; [2] tested range concentration: 2.5 - 0.039 μg/mL; [3] n.d., not determined

Table 2. Percentage values of cell viability and hemolytic activity at 100 μM (mean and standard deviation).

Compound	HEL 299	hRBCs
**1a**	62.26 ± 5.42	< 5
**1b**	6.72 ± 0.80	< 5
**2a**	1.07 ± 0.31	< 5
**2b**	1.48 ± 0.15	< 5
**3**	0.82 ± 0.19	< 5
**CP**[1]	0.36 ± 0.11	n.d.[2]
**Triton X-100**[3]	n.d.	100 ± 2.6

[1] CP, cisplatin at 166.6 μM; [2] n.d., not determined; [3] Triton X-100 at 1%.

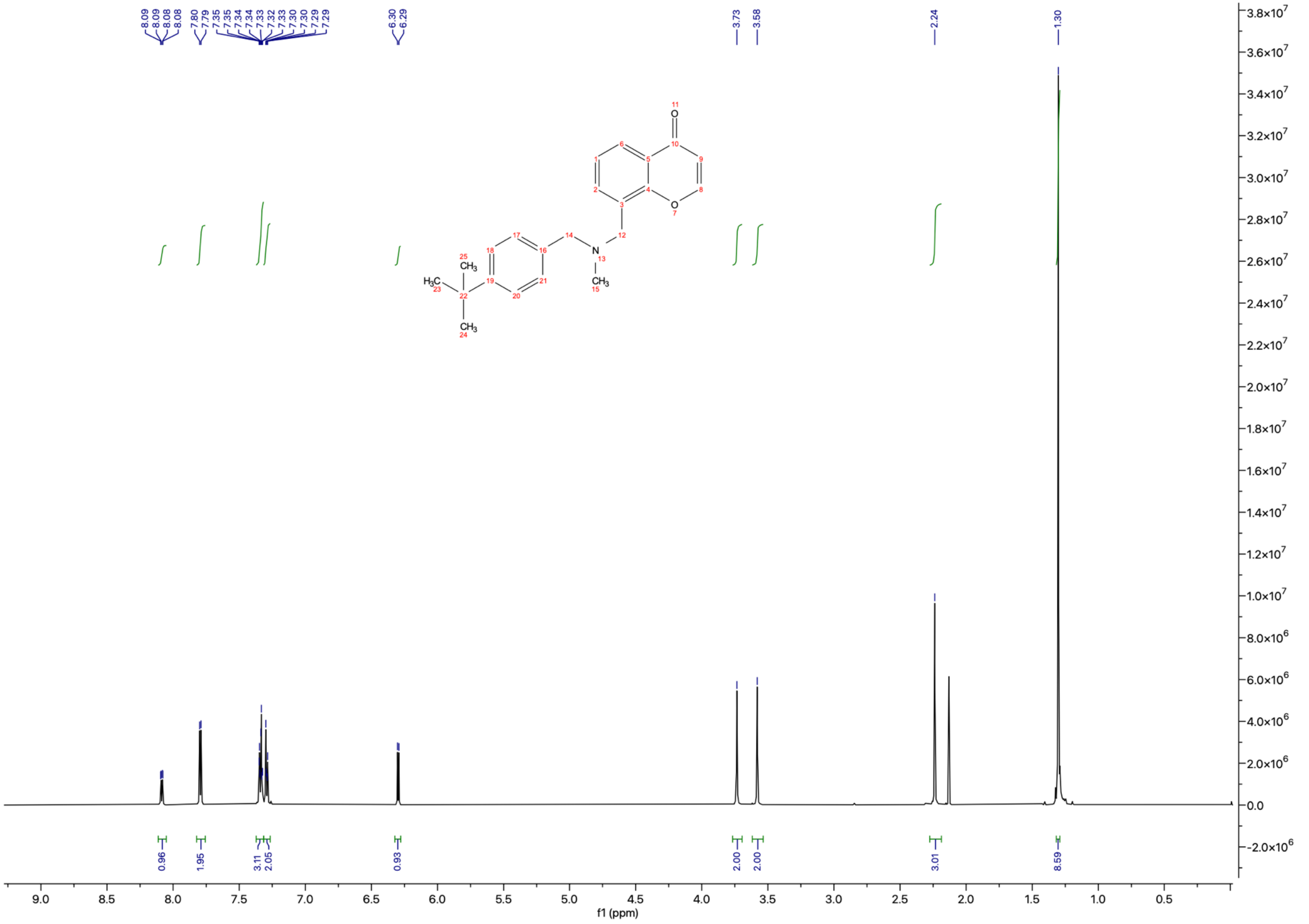

Figure S1: ^{1}H-NMR Spectra of compound **1a**

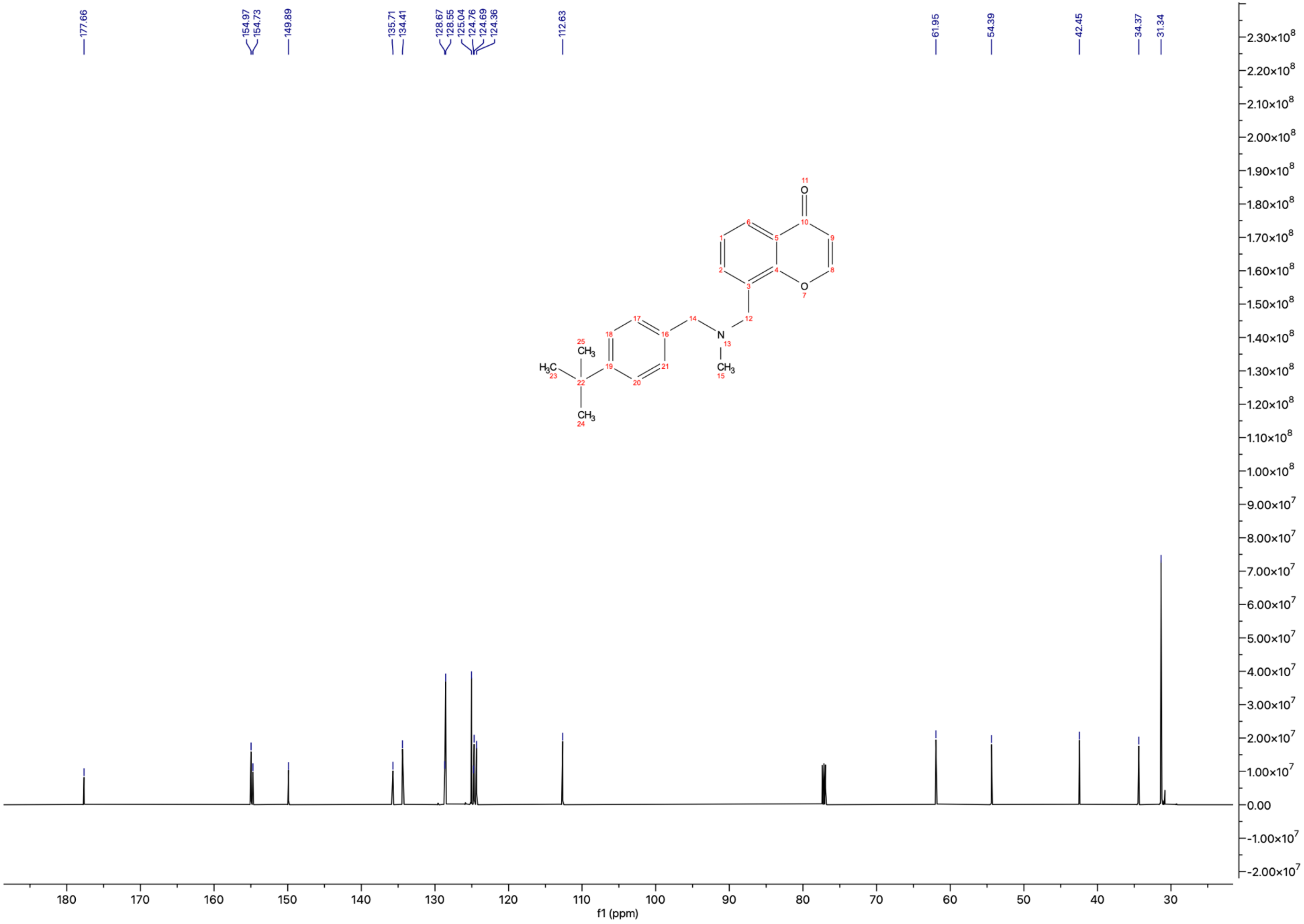

Figure S2: ^{13}C-NMR Spectra of compound **1a**

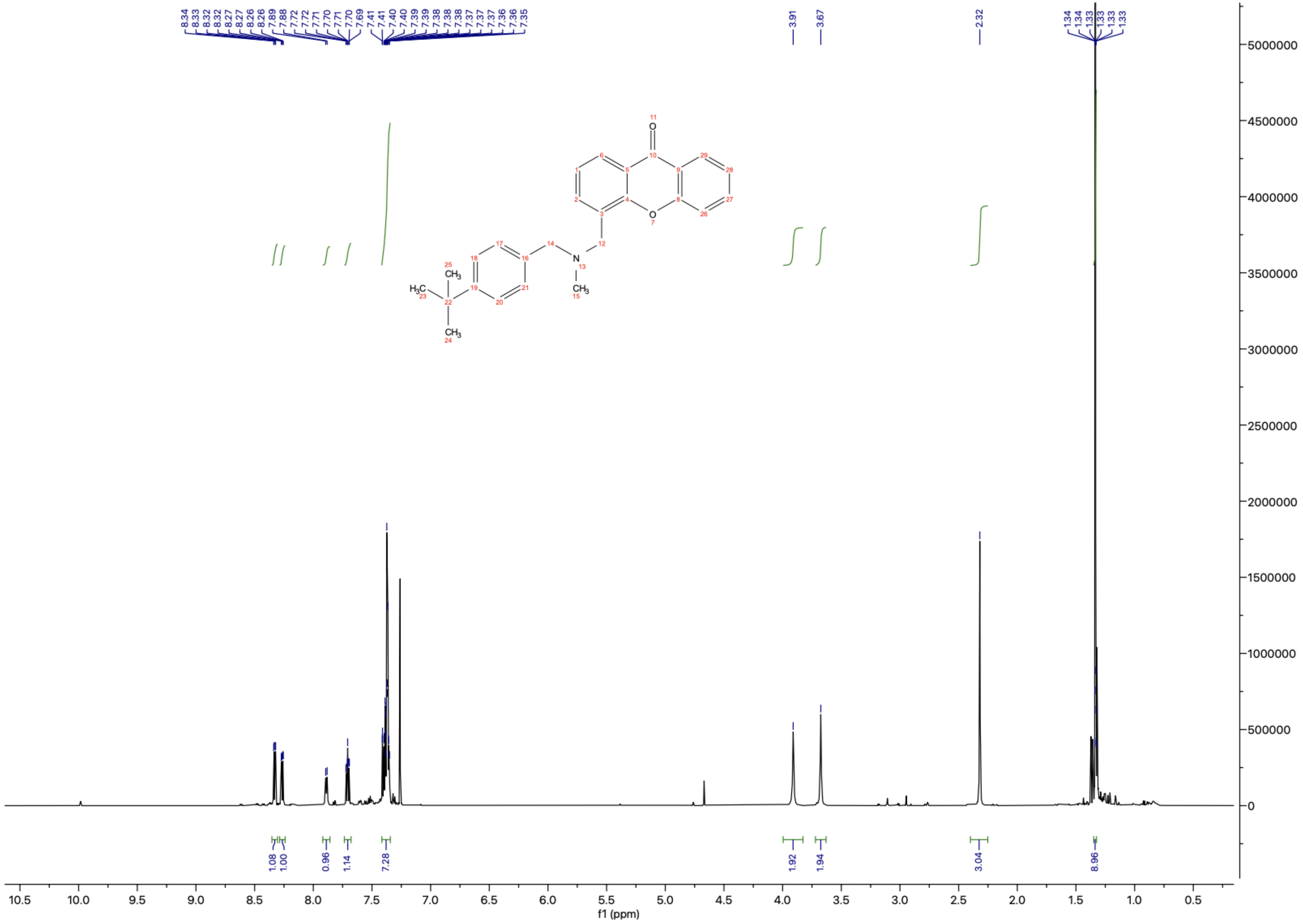

Figure S3: ^{1}H-NMR Spectra of compound **1b**

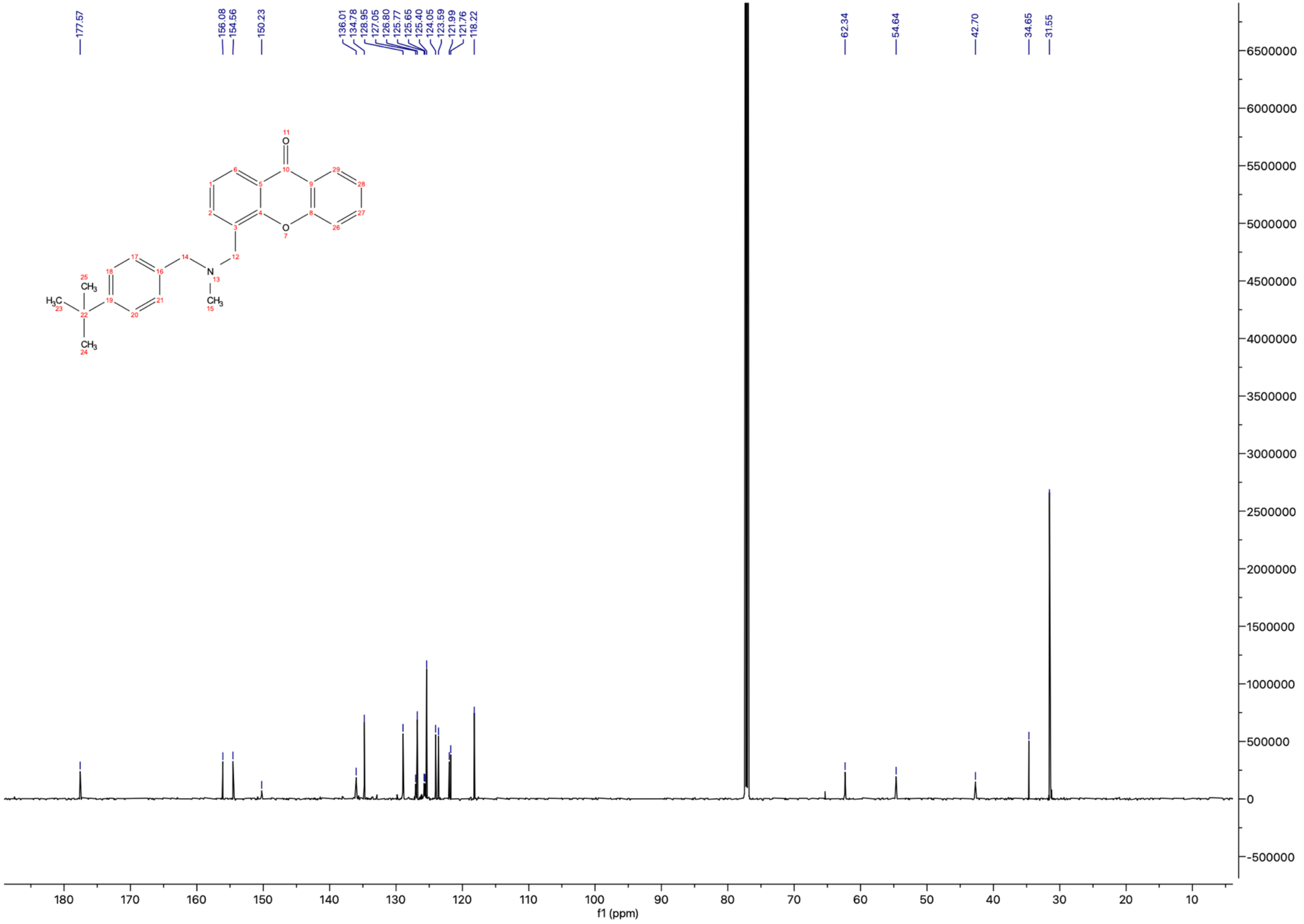

Figure S4: ^{13}C-NMR Spectra of compound **1b**

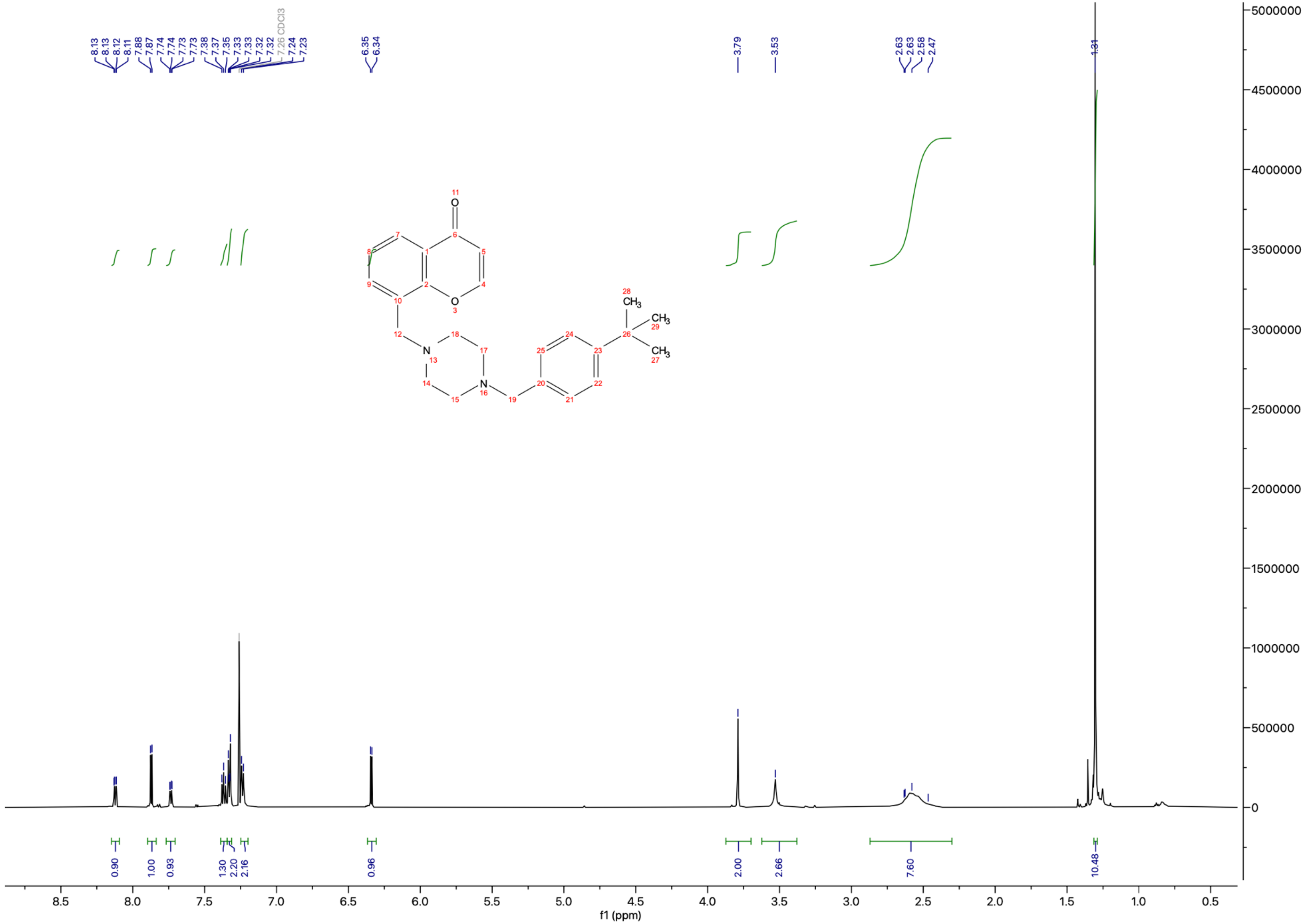

Figure S5: ^{1}H-NMR Spectra of compound **2a**

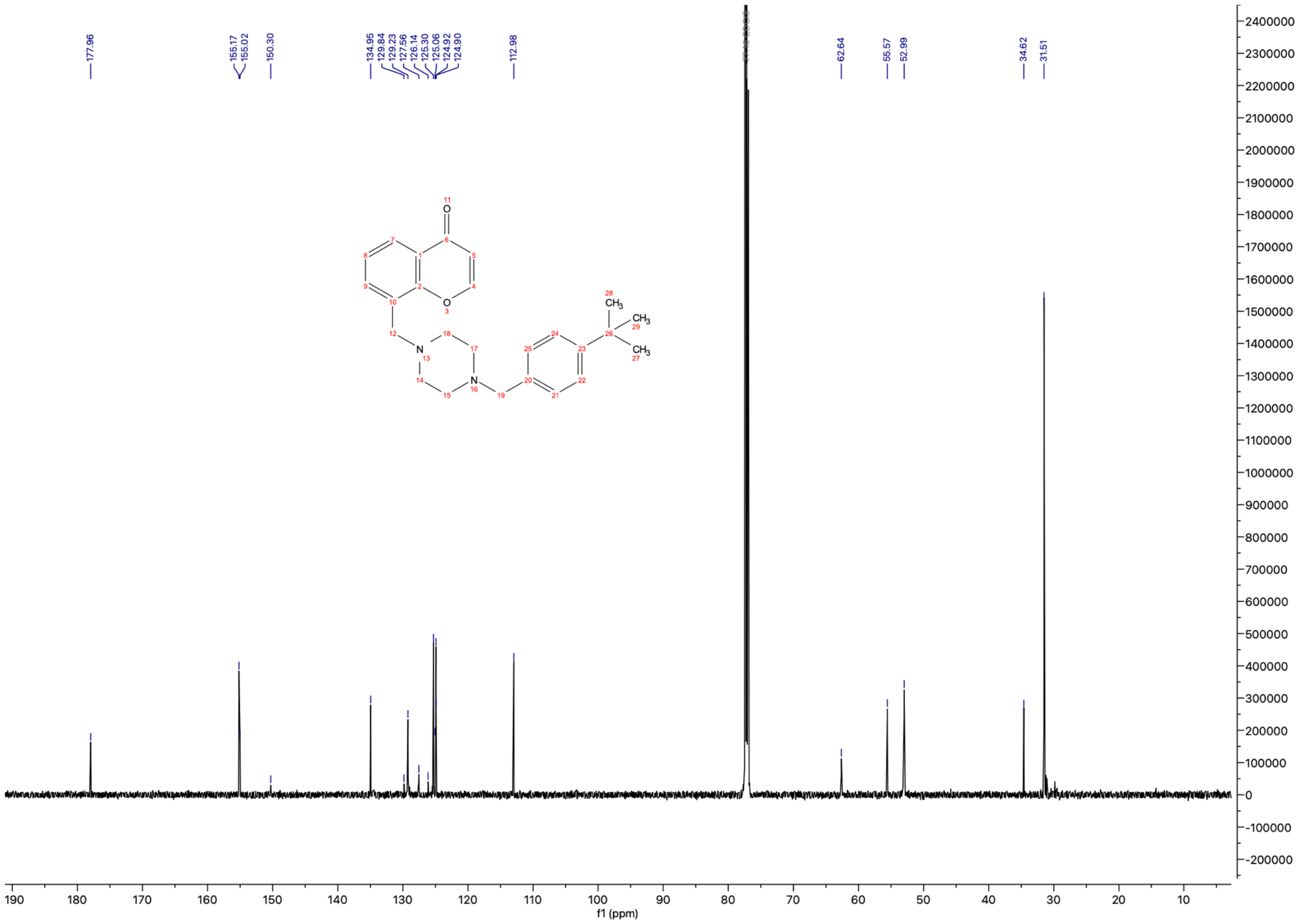

Figure S6: ^{13}C-NMR Spectra of compound **2a**

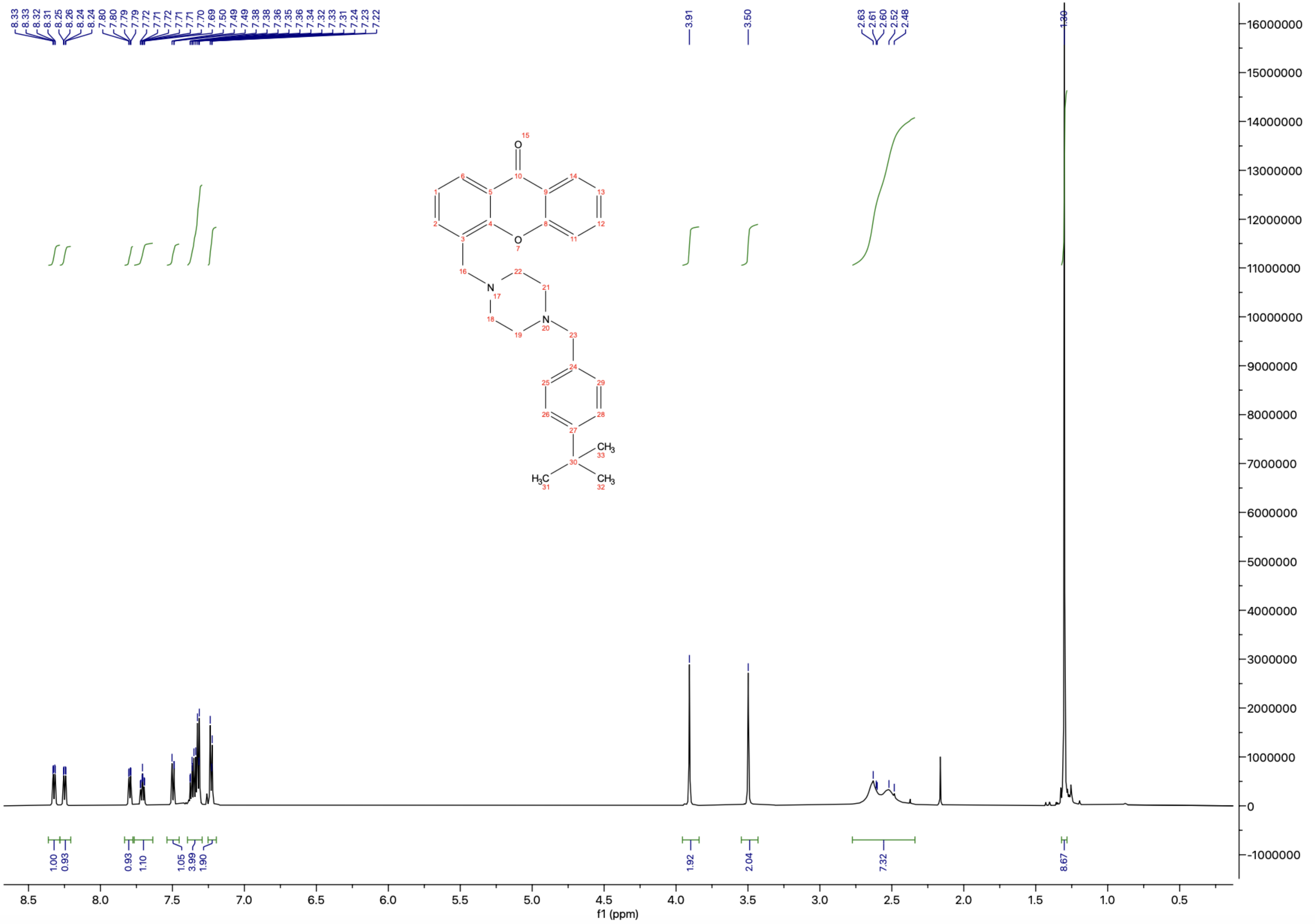

Figure S7: ^{1}H-NMR Spectra of compound **2b**

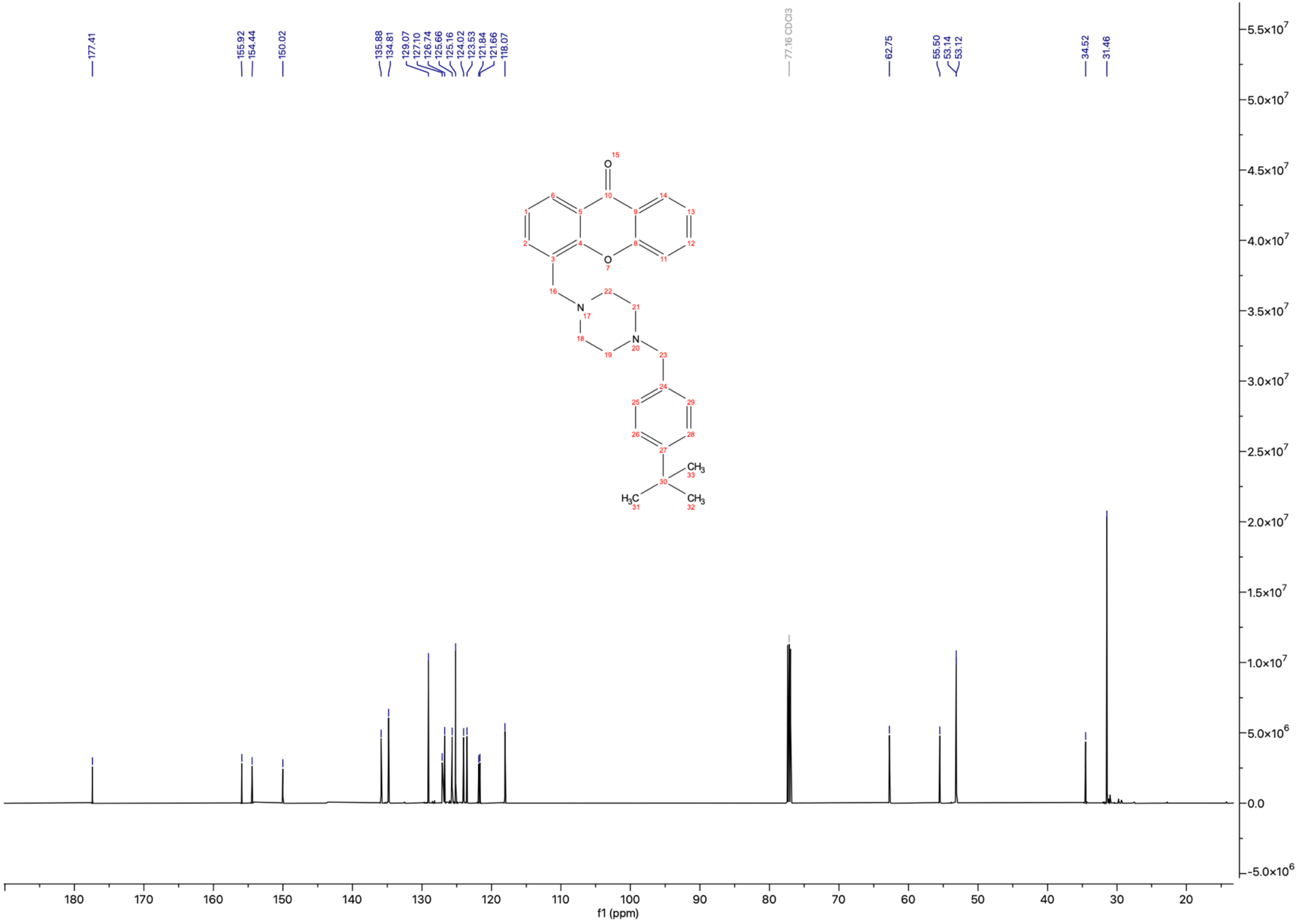

Figure S8: ^{13}C-NMR Spectra of compound **2b**

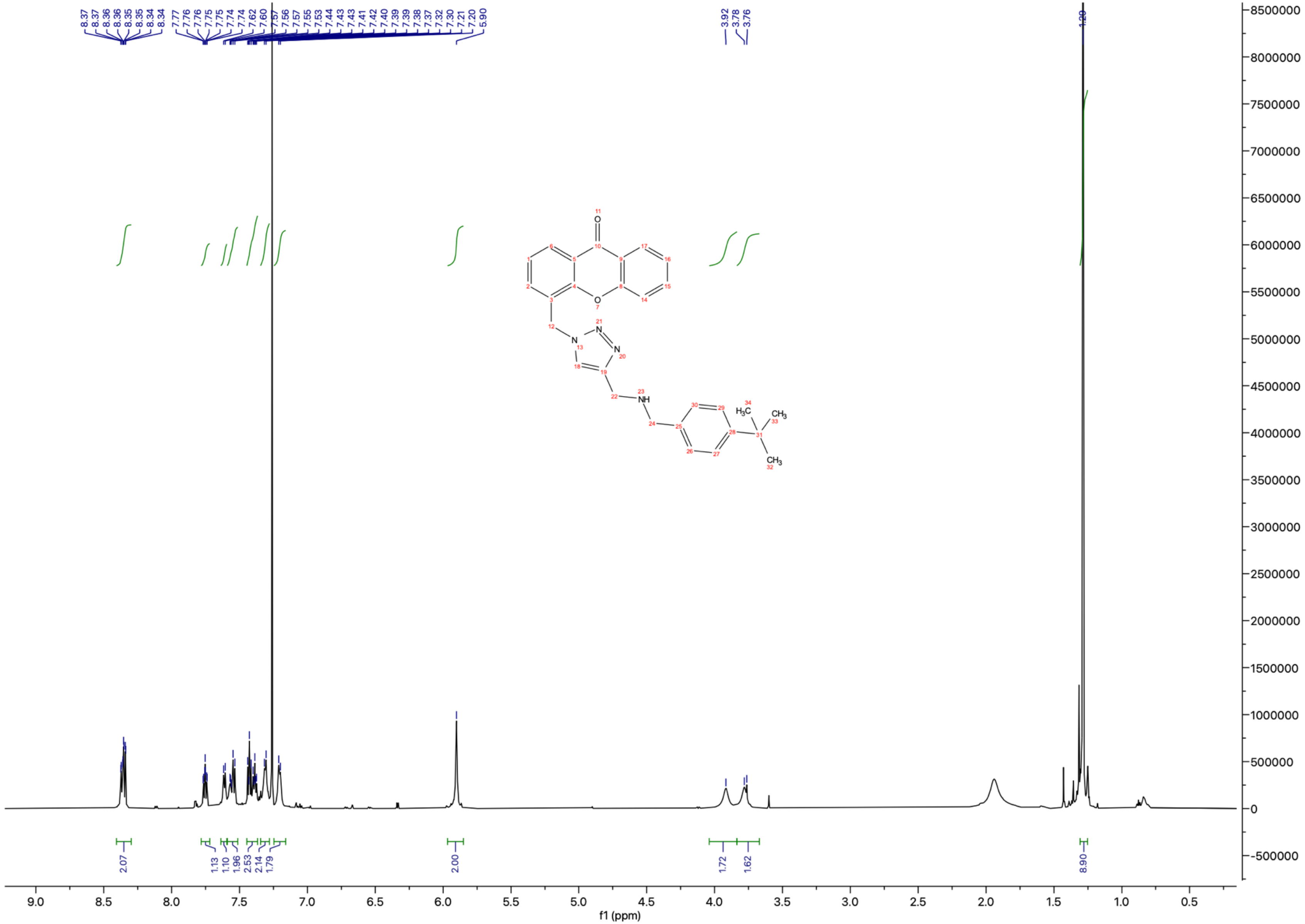

Figure S9: ^{1}H-NMR Spectra of compound **3**

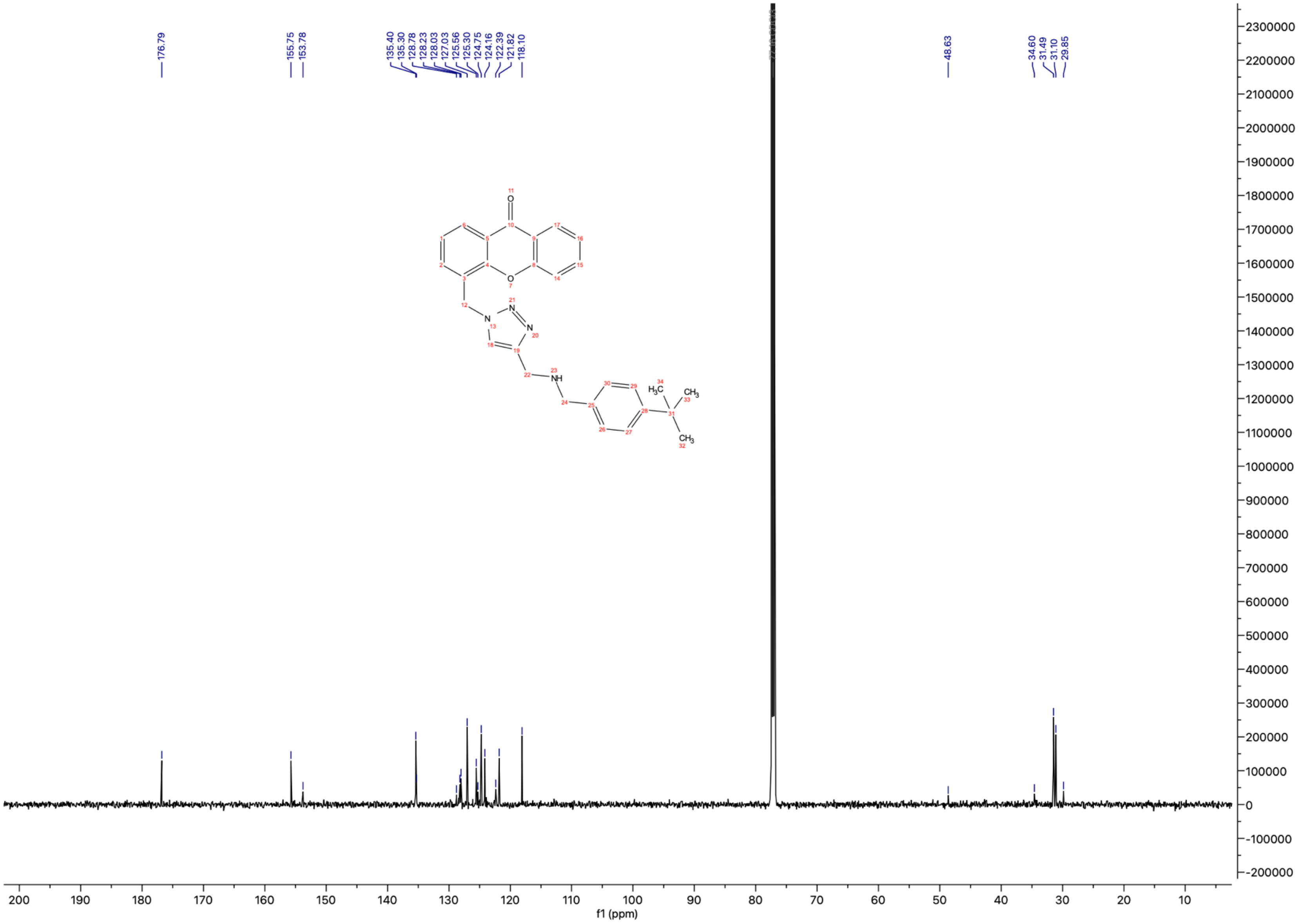

Figure S10: ^{13}C-NMR Spectra of compound **3**

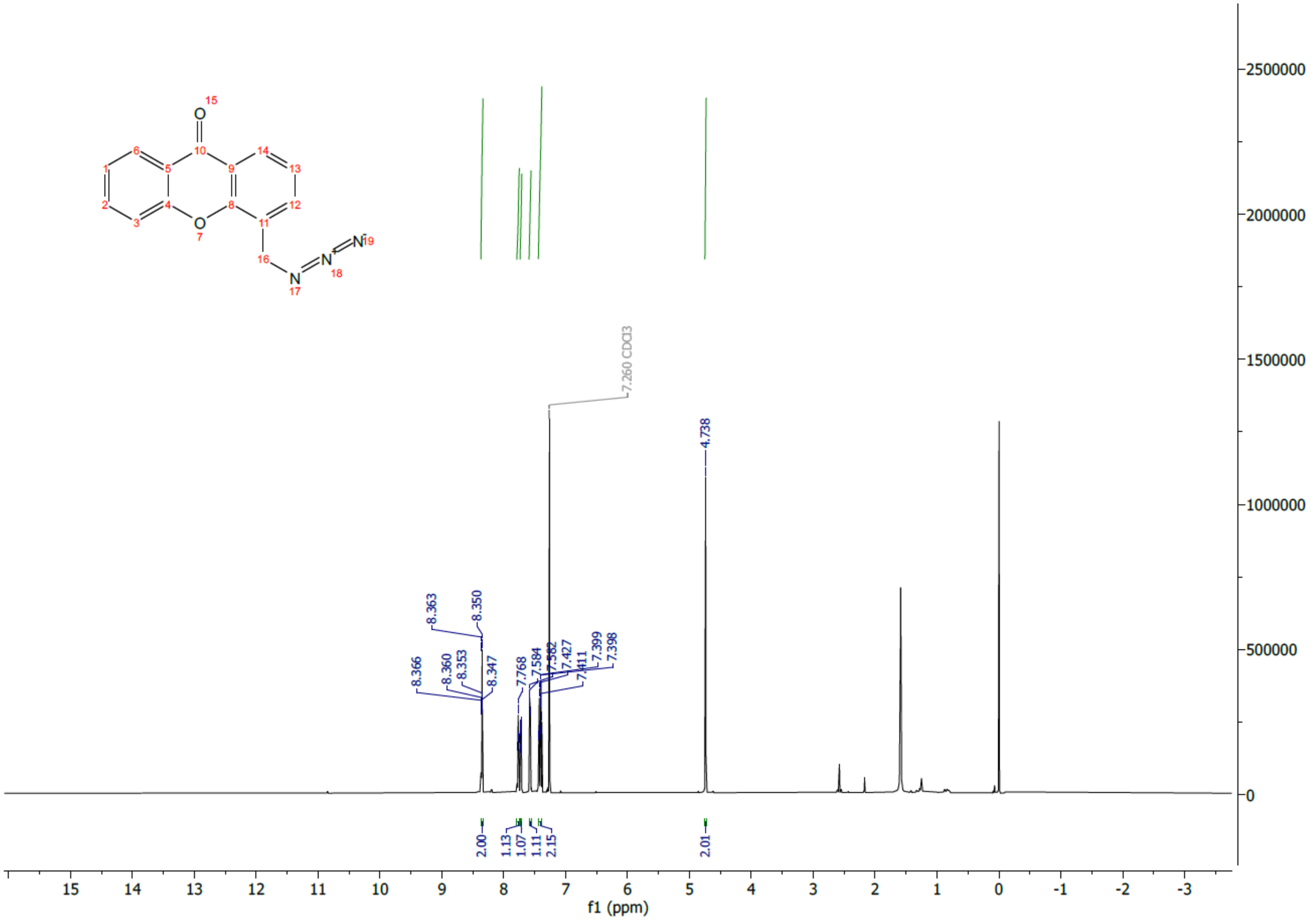

Figure S11: ^{1}H-NMR Spectra of intermediate **8**

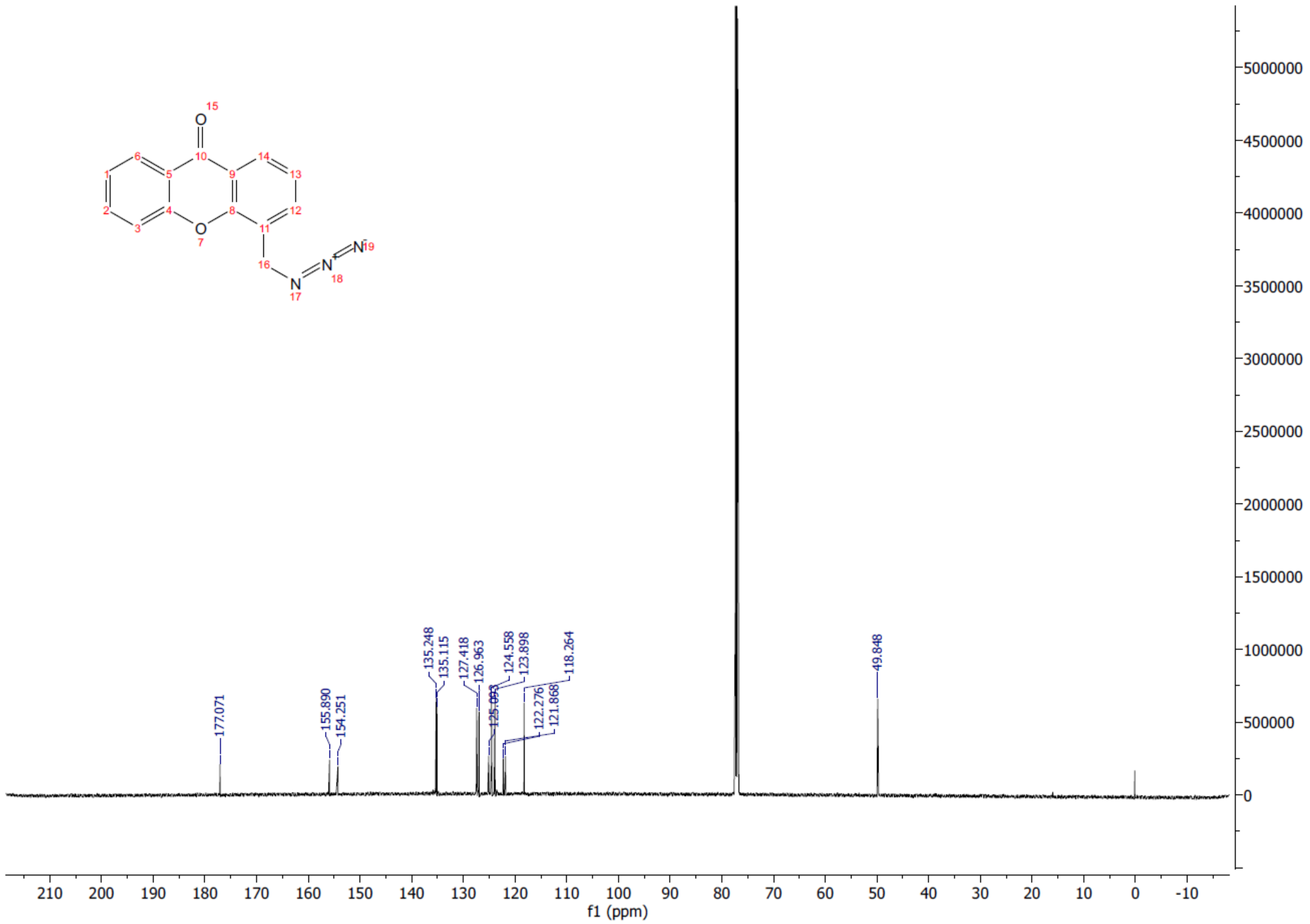

Figure S12: ^{13}C-NMR Spectra of intermediate **8**

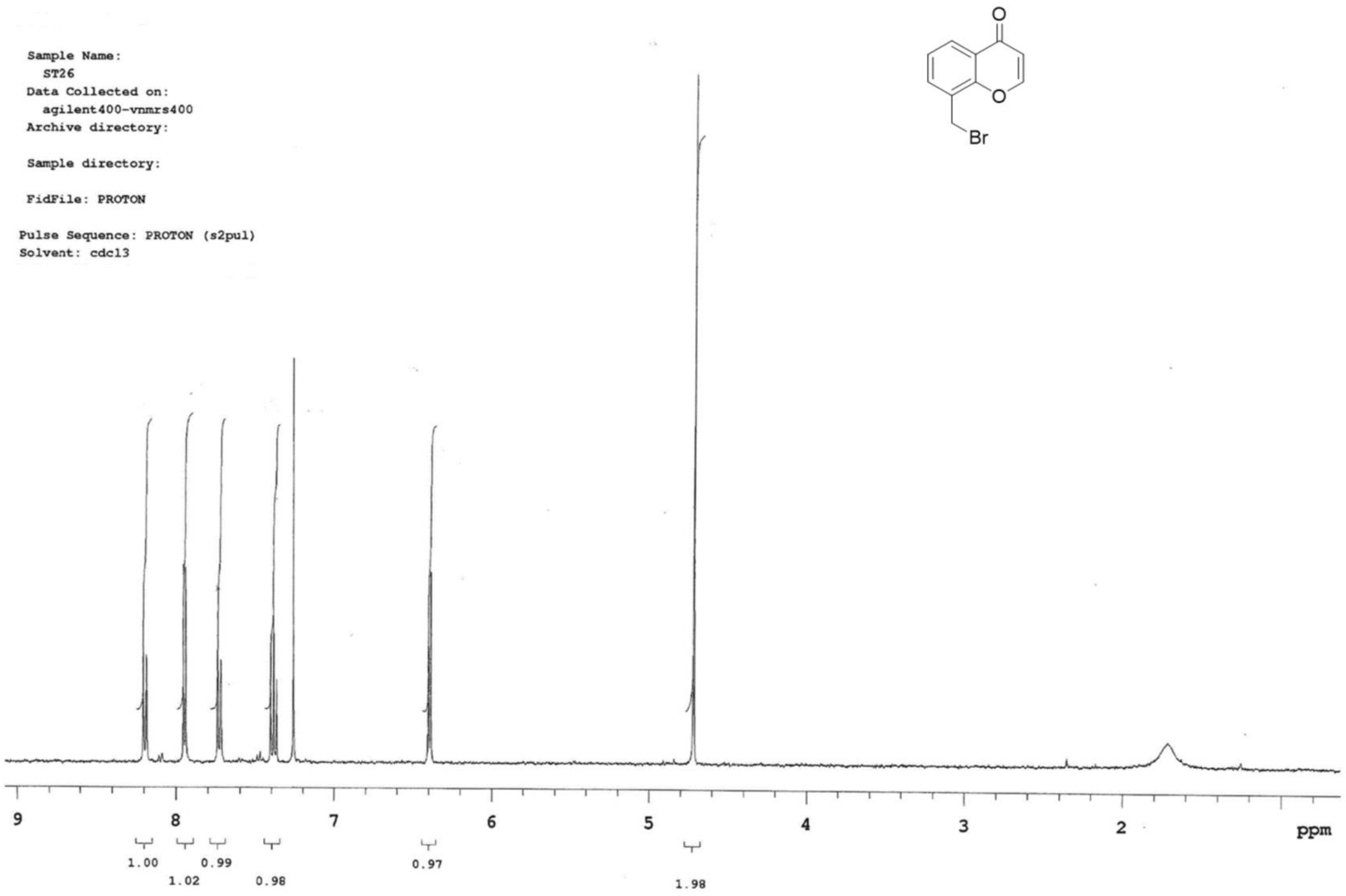

Figure S13: ^{1}H-NMR Spectra of intermediate **6**
